# Diffusion-Weighted Imaging in the Musculoskeletal System: Evolving Role in Modern Imaging Practice

**DOI:** 10.3390/diagnostics16162622

**Published:** 2026-08-18

**Authors:** Ankit Tandon, Gurukrishna Bindhumadhavan

**Affiliations:** 1 Department of Radiology, Tan Tock Seng Hospital, Singapore 308433, Singapore; b.gurukrishna20@gmail.com; 2 Lee Kong Chian School of Medicine, Nanyang Technological University, Singapore 308232, Singapore

**Keywords:** diffusion-weighted imaging (DWI), musculoskeletal system, apparent diffusion coefficient (ADC), tumour composition, tumour characterisation, myxoid tumours, chondroid tumours, multiparametric MRI, osteomyelitis, soft tissue abscess, whole-body MRI, treatment response, vertebral fractures, pathological fractures, deep vein thrombosis, DWI pitfalls

## Abstract

Diffusion-weighted imaging (DWI) has evolved from a niche research sequence into an increasingly valuable adjunct to conventional magnetic resonance imaging (MRI) in musculoskeletal (MSK) radiology. By providing qualitative and quantitative information on tissue microstructure through assessment of water diffusion and apparent diffusion coefficient (ADC) mapping, DWI offers functional insights beyond conventional morphological imaging. We aim to present the current evidence for DWI in MSK imaging organised around established applications and emerging applications, with particular emphasis on composition-related interpretive pitfalls relevant to differentiating tumours and other pathologies, and to review the technique’s evolving role in routine practice. This narrative review synthesises the current literature on the clinical utility of DWI in MSK imaging. It is structured in four parts: foundations and the tissue composition signal framework, including the basis of qualitative and quantitative assessment; established applications; emerging applications; and assessment of tissue composition-related interpretive as well as technical pitfalls, including those arising due to myxoid matrix, chondroid matrix, blood degradation products, organising thrombus, crystalline or mineralised material, keratinaceous debris, purulent content, cellular haematopoietic marrow, by using original cases from the authors’ institution, which have been confirmed either histologically or surgically. Applications are stratified by strength of evidence. Established applications of DWI include soft tissue abscess detection, differentiation of malignant from benign soft tissue tumours, differentiation of malignant from benign vertebral compression fractures, and myeloma staging and response assessment, as well as treatment response in soft tissue and bone sarcomas. Whole-body MRI with DWI for staging and response assessment in multiple myeloma is guideline-endorsed and supported by prospective multicentre data. Soft tissue abscess detection, soft tissue and bone tumour characterisation, and characterisation of vertebral compression fractures are supported by consistent evidence from multiple independent cohorts, although no universally transferable ADC threshold exists. The emerging applications, which are promising adjuncts supported by small, single-centre or heterogeneous studies with thresholds that have not been externally validated, include ADC ghost sign in osteomyelitis (high specificity but sensitivity of only 20%), peripheral nerve sheath tumour characterisation and surveillance in NF1 patients, peripheral neuropathy and plexopathy, predisposing conditions such as Li Fraumeni syndrome in paediatric cancers, inflammatory myopathy, and postsurgical assessment of residual disease, as well as opportunistic detection of venous thrombosis. Radiomics and machine learning approaches remain experimental. Recent technical advances, including reduced field-of-view imaging, multi-shot acquisition and improved fat suppression, have mitigated but not eliminated historical limitations of susceptibility artefacts and limited spatial resolution. DWI has become an important functional imaging technique that complements conventional MRI across a broad range of musculoskeletal disorders. Understanding the relationship between tissue composition and the diffusion signal is central to both interpreting DWI correctly and avoiding its characteristic pitfalls. DWI is best regarded not as a stand-alone technique but as one component of a multiparametric assessment, in which its functional information is integrated with conventional morphological imaging. Ongoing technical improvement and expanding clinical evidence are expected to further support its integration into routine MSK imaging and its development as a quantitative biomarker for diagnosis, prognostication, and treatment monitoring.

## 1. Background and Introduction

Diffusion-weighted imaging (DWI) is an MRI sequence that characterises tissue at the microstructural level by measuring the Brownian motion of water molecules within biological tissues [1,2]. Originally a cornerstone of neurological imaging, most notably for detecting acute cerebral infarction, DWI has progressively migrated into musculoskeletal (MSK) radiology, where it now plays a central and expanding role in diagnostic strategy and therapeutic monitoring [3,4].

In an unrestricted environment, water molecules diffuse randomly in all directions, a state termed isotropic diffusion. Within biological tissues, however, the complex internal architecture comprising cell membranes, tight junctions, and macromolecules impede this random motion, producing restricted or anisotropic diffusion [1,2]. Regions exhibiting restricted diffusion, such as those with high cellularity or purulent collections, appear hyperintense on DWI sequences, while areas of free diffusion, such as extracellular fluid and normal blood vessels, appear hypointense. This differential signal behaviour of DWI can significantly enhance the diagnostic capability of conventional MRI [3].

The well-organised collagen architecture of MSK connective tissues comprising tendons, ligaments, peripheral nerves, and cartilage make them particularly amenable to diffusion-based evaluation, as structural deterioration alters diffusion characteristics in measurable and diagnostically meaningful ways [5,6]. The apparent diffusion coefficient (ADC), the primary quantitative metric derived from DWI, provides a numerical reflection of tissue cellularity and water mobility, enabling objective lesion characterisation. Advanced extensions, including diffusion tensor imaging (DTI) and diffusion kurtosis imaging (DKI), yield additional parameters such as fractional anisotropy (FA), mean diffusivity (MD), and mean kurtosis (MK), providing deeper insight into tissue microstructure and fibre architecture [6,7].

Existing reviews have comprehensively catalogued the reported applications of DWI in the musculoskeletal system and its incremental value over conventional MRI [3,4,8]. The present review examines the foundations of DWI in MSK imaging through a tissue-composition-based signal framework. It also discusses established and emerging clinical applications, with particular emphasis on interpretative and technical pitfalls related to tissue composition. A tissue-composition-based interpretative framework is essential because the diagnostic yield of DWI in MSK imaging depends not only on tissue cellularity but also on the microenvironment, which may impede or facilitate the movement of water molecules. Furthermore, the predominant physical substrate of a lesion generates a characteristic and often predictable diffusion signal, which can provide valuable diagnostic clues and assist in lesion characterisation. Myxoid matrix, chondroid matrix, hemosiderin, evolving blood degradation products, crystalline and mineralised material, fibrous content, keratinaceous and cholesterol-rich debris, purulent content, cellular haematopoietic marrow, and organising thrombus each impose characteristic constraints on water mobility and on intrinsic T2 relaxation. The consequences of these constraints, which have been historically catalogued as “pitfalls”, when read as compositional evidence within the multiparametric MRI assessment framework, are among the most specific information DWI provides.

This framework is developed in Section 3, as summarised in Section 3, and is then applied across established applications (Section 4, Section 5 and Section 6), emerging applications (Section 7, Section 8, Section 9 and Section 10), and, in detail, the interpretive scenarios in which composition dominates the signal (Section 12). Throughout, the argument is illustrated by original cases from our institution, confirmed histologically or surgically. We have deliberately retained cases in which DWI performed poorly, such as a malignant myxoid tumour without restriction, a benign cyst with convincing restriction, a gouty tophus with spuriously low ADC in the absence of DWI hyperintensity, hyperplastic red marrow mimicking myelomatous infiltration and similar presentations, because such discordant cases are the substrate of the framework and are comparatively underrepresented in the published literature. Technical considerations, artefact mitigation and the future role of DWI as a multiparametric biomarker are addressed where they bear on this argument.

*What this review adds.* Existing reviews establish what DWI can detect in the musculoskeletal system [3,4,8]. This review addresses that question but also asks how the signal should be read when it appears to mislead. It (i) proposes an explicit mapping between lesion composition and DWI/ADC behaviour and applies it consistently across applications and pitfalls (Section 3); (ii) grades every application by evidentiary maturity so that guideline-endorsed uses are not presented alongside single-centre observations as though they carried equal weight (Section 3.2); (iii) illustrates each compositional mechanism with original, histologically or surgically confirmed institutional cases, deliberately including discordant examples in which DWI performed poorly; and (iv) describes the opportunistic detection and ageing of deep vein thrombosis on routine limb MSK DWI, an incidental finding with direct therapeutic consequences that is not covered in the cited reviews.

### Evidence Base and Search Strategy

This is a narrative rather than a systematic review, and no PRISMA protocol was registered. PubMed/MEDLINE, Embase, Scopus and the Cochrane Library were searched from January 2008 to December 2025, using combinations of the terms “diffusion-weighted imaging”, “DWI”, “apparent diffusion coefficient”, “ADC”, “diffusion tensor imaging”, “DTI” and “diffusion kurtosis imaging”, combined with musculoskeletal terms including “soft tissue tumour”, “sarcoma”, “bone tumour”, “bone marrow”, “multiple myeloma”, “whole-body MRI”, “vertebral compression fracture”, “osteomyelitis”, “abscess”, “septic arthritis”, “inflammatory myopathy”, “peripheral nerve sheath tumour”, “plexopathy”, “deep vein thrombosis” “treatment response”, “MSK applications of DWI” and “pitfalls in DWI”. Reference lists of retrieved articles and relevant ESSR, MY-RADS and MET-RADS-P consensus documents were hand-searched. Only English-language publications were considered. Titles and abstracts were screened by initials; full texts were assessed for original musculoskeletal DWI data, technical validation, or formal consensus guidelines. Case reports and conference abstracts were excluded, except where used solely for illustrative purposes. Landmark studies were also included when they introduced important concepts or remain foundational to current clinical practice, and additional relevant references were identified through citation tracking. Where reported findings were inconsistent, we gave precedence in the following order: systematic reviews and meta-analyses; international consensus and guideline documents; prospective studies with an independent histological, microbiological or surgical reference standard; and larger or more recent retrospective single-centre series. Where the available evidence was limited to small retrospective series, this is stated explicitly in the text, and the application is described as emerging rather than established. Illustrative figures are drawn from the authors’ institutional practice and are presented as teaching examples rather than as evidence. The evidence base underpinning the principal applications is summarised in Table 1. Generative AI (Claude, Anthropic) was used to assist with reorganising the manuscript, drafting and refining connective and summary text, and editorial renumbering and formatting; all such output was reviewed and verified by the authors.

## 2. Technical Considerations

### 2.1. DWI Acquisition Principles

**Figure 1:**
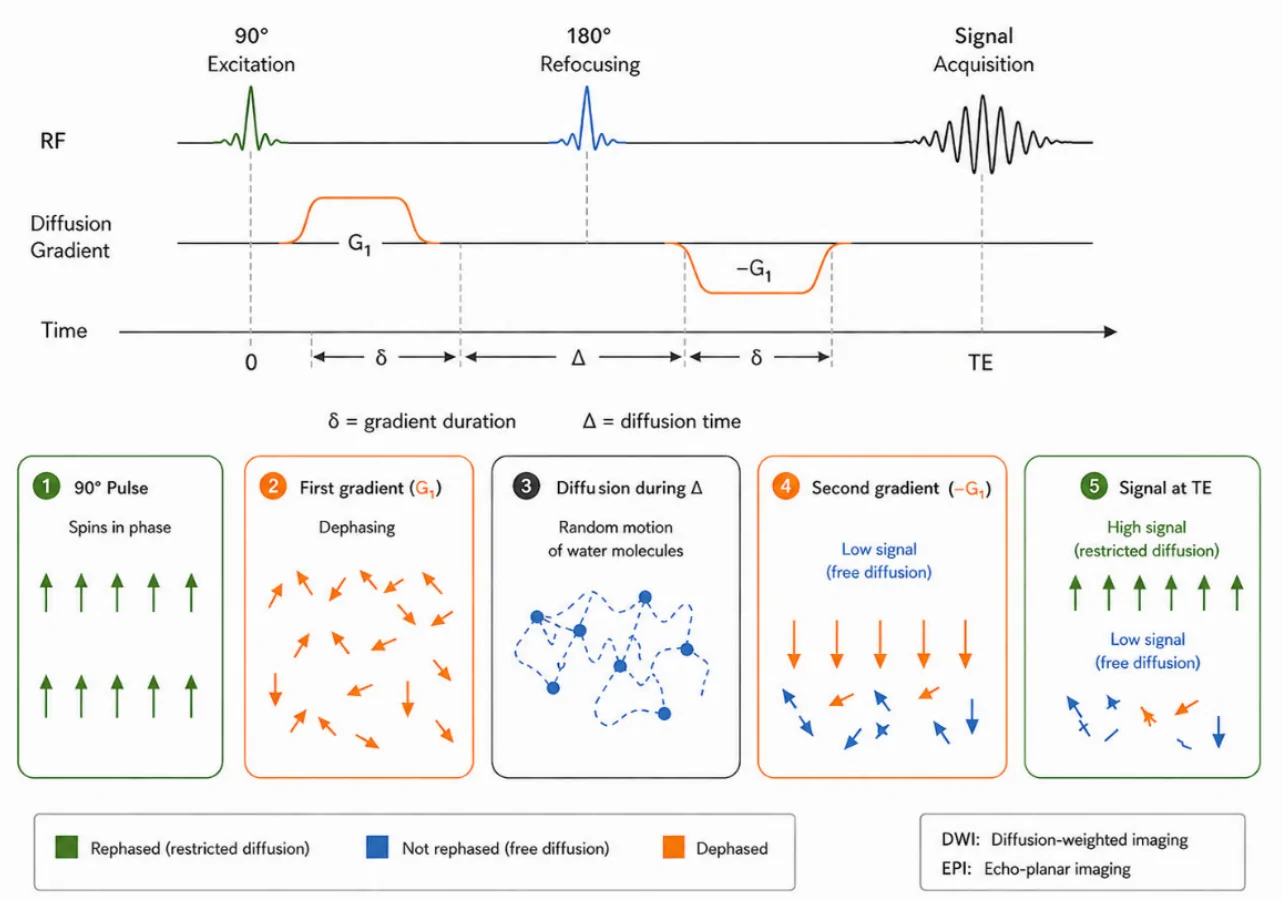
DWI measures water diffusivity by applying two diffusion-sensitising gradients in equal but opposite directions. If the molecules have moved before the second gradient, rephasing is incomplete and signal loss occurs (dark on DWI). The reverse occurs if water molecules are restricted; they retain bright signal.

Diffusion-weighted images are most commonly acquired using a diffusion-weighted spin-echo sequence with an echo-planar imaging (EPI) readout, to which a pair of diffusion-sensitising gradients is added, usually combined with fat suppression (Figure 1). Because of its long echo time, the sequence retains substantial intrinsic T2 weighting, which is the origin of the T2 shine-through and T2 black-out effects discussed in Section 12; it is not, however, a conventional fat-suppressed T2-weighted sequence, and the two should not be equated.

The technique relies on the application of two equal and opposite diffusion gradients placed on either side of a 180° refocusing pulse [38]. Following an initial 90° radiofrequency excitation pulse, the first gradient induces phase dispersion (dephasing) of proton spins. During the subsequent time interval, water molecules undergo diffusion. A second, opposing gradient is then applied to rephase the spins.

In tissues where water diffusion is restricted (e.g., highly cellular lesions), proton displacement is minimal, allowing effective rephasing and resulting in high signal intensity on DWI. In contrast, in areas of free diffusion, significant proton movement occurs between the gradients, preventing complete rephasing and leading to signal attenuation [38,39] (Figure 1).

The degree of diffusion weighting is determined by the b-value, with higher b-values increasing sensitivity to diffusion restriction.

In current clinical MSK practice, diffusion-weighted imaging is predominantly acquired using single-shot echo-planar imaging (ssEPI), which captures the entire k-space in a single acquisition, minimising motion artefacts and enabling rapid data collection [39,40]. While practical, single-shot EPI is prone to magnetic susceptibility artefacts at tissue interfaces, particularly air–bone–soft tissue boundaries, as well as geometric distortions and lower spatial resolution. Multi-shot EPI divides k-space across multiple excitations, achieving superior spatial resolution and reduced geometric distortion, although at the cost of increased acquisition time, making it susceptible to motion artefacts. Furthermore, more sophisticated phase-correction algorithms are required to compensate for inter-shot bulk motion [40].

### 2.2. b-Values and Signal Behaviour

The b-value is an acquisition parameter reflecting the strength, duration and temporal separation of diffusion-sensitising gradients [38]. Under a monoexponential model, the ADC is obtained from the negative slope of the natural logarithm of signal intensity plotted against the b-value; acquiring more than two b-values improves the precision of the ADC calculation. Higher b-values increase sensitivity to diffusion restriction but reduce the signal-to-noise ratio, and at high b-values, the monoexponential assumption itself becomes less secure as non-Gaussian diffusion effects emerge [38].

**Figure 2:**
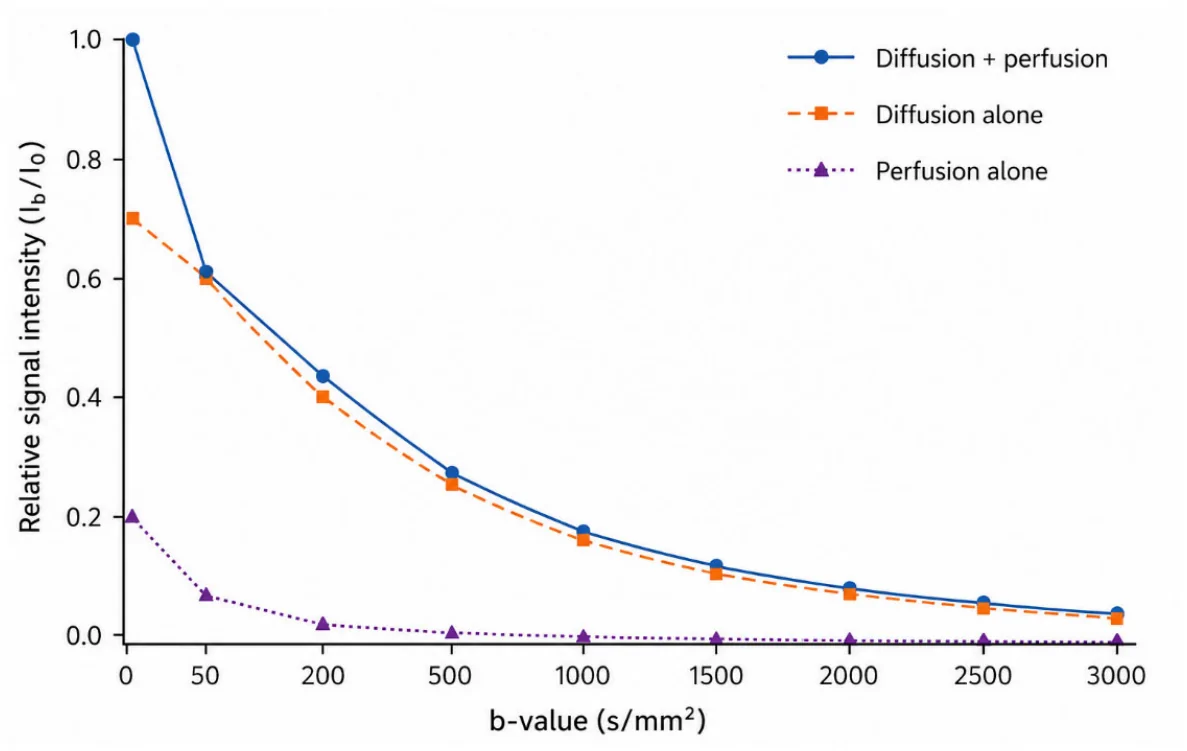
b-values in DWI indicate the strength and duration of the diffusion-sensitising gradients. In the graph, b-values are plotted on the *x*-axis and signal intensity on the *y*-axis. As the b-value increases, signal intensity decays exponentially. At lower b-values, the perfusion-related contribution is proportionally greater, so a fit including b = 0 will overestimate diffusivity; omitting b = 0 reduces this contribution but does not remove it, and the resulting measurement remains an apparent diffusion coefficient, still influenced by residual perfusion, noise and non-Gaussian diffusion. As b-values increase beyond approximately 1000 s/mm^2^, the signal in most musculoskeletal tissue approaches the noise floor. At our institution, we use three b-values (50, 400 and 800 s/mm^2^) for musculoskeletal imaging, omitting b = 0 to limit the perfusion contribution and avoiding b-values above 1000 s/mm^2^ because of low signal-to-noise ratios. This is one reasonable choice among several b-value selections.

In musculoskeletal diffusion-weighted imaging (DWI), signal behaviour is strongly dependent on the selected b-values. In the absence of diffusion sensitising gradients (b = 0 s/mm^2^), images are predominantly T2-weighted, and free water appears bright due to T2 shine-through (Figure 2). Consequently, b = 0 images closely resemble fat-suppressed T2-weighted sequences and are significantly influenced by perfusion, which can lead to overestimation of apparent diffusion coefficient (ADC) values. For this reason, many contemporary MSK protocols exclude b = 0 and instead use a low non-zero b-value (typically ≈50 s/mm^2^) to reduce perfusion-related contributions while preserving the signal.

At low b-values (≈50–100 s/mm^2^), perfusion effects are still present but reduced compared to b = 0. Fast-flowing fluids, such as blood within vessels, show signal attenuation, producing a “black-blood” effect. At this stage, images still retain substantial T2 weighting. As the b-value increases, signal intensity progressively decreases across tissues, with the most rapid attenuation seen in free water and fluid collections, followed by less organised or glandular tissues.

Intermediate b-values (≈100–300 s/mm^2^) represent a transition zone where perfusion influence reduces and true diffusion effects begin to dominate. High b-values (≥500–800 s/mm^2^) are essential in MSK imaging to further suppress perfusion-related signals and better reflect true water diffusion within tissues. In this range, highly cellular lesions such as malignant tumours demonstrate restricted diffusion and remain relatively hyperintense against a progressively darkening background. However, it is important to note that some normal highly cellular tissues (e.g., central nervous system structures, spleen, and lymphatic tissue) may also appear relatively hyperintense at high b-values.

An optimal b-value range of approximately 500–800 s/mm^2^ is widely considered appropriate in MSK imaging, as it minimises perfusion effects while maintaining an adequate signal-to-noise ratio (SNR). The use of very high b-values (>1000 s/mm^2^) is generally not recommended in routine MSK practice, as they significantly reduce the SNR without consistently improving diagnostic accuracy.

There is currently no universal consensus regarding the ideal number and selection of b-values in musculoskeletal imaging. Earlier studies employed multiple b-values, which increased scan time without proportionate diagnostic benefit; one such study evaluated DWI using four b-values (0, 300, 800 and 1400 s/mm^2^) and found comparable diagnostic accuracy between simplified and more complex combinations at longer acquisition times [41]. Consequently, most contemporary protocols favour a limited number of b-values, commonly three (for example, 50, 400 and 800 s/mm^2^ [3,8], as also used at our institution), to balance acquisition time, image quality and quantitative reliability. Whole-body protocols differ deliberately: as described in Section 5.1, our whole-body skeletal survey uses b = 0 and 1000 s/mm^2^. The larger field of view, lower spatial resolution and greater need for background suppression in whole-body imaging favour a high upper b-value for lesion conspicuity and inverted-MIP reconstruction, and the two-point acquisition keeps whole-body scan time tolerable; the trade-off is a coarser and more perfusion-influenced ADC estimate than the alternate protocol provides.

### 2.3. ADC Quantification and Region of Interest Considerations

**Figure 3:**
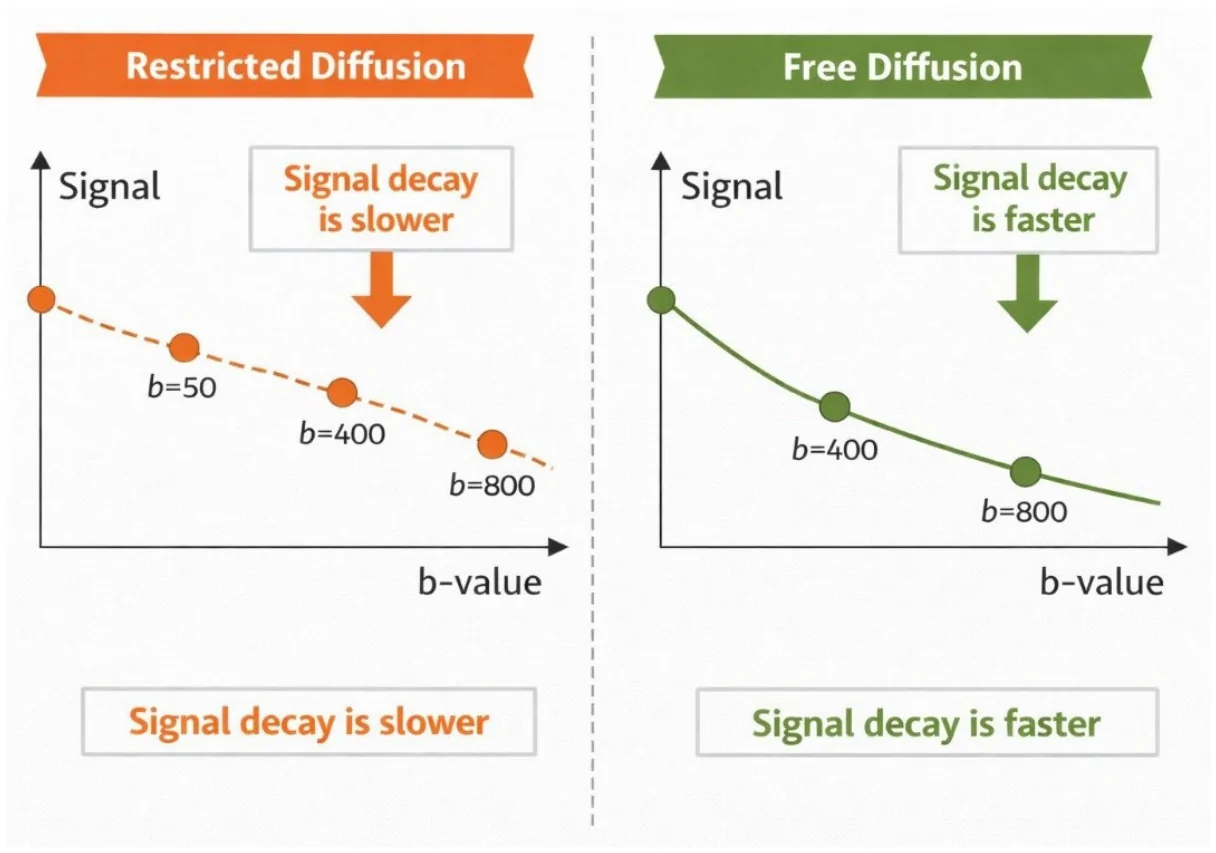
ADC represents the rate of diffusion-related signal attenuation as the b-value increases. Two or more b-values are selected to measure the rate of decay. If water moves freely, the rate of signal decay between b-values is faster, reflected as high ADC values. If water motion is restricted, the rate of signal decay is slower, reflected as low ADC values.

The apparent diffusion coefficient (ADC) is a pixel-by-pixel quantitative measure that combines more than just true molecular diffusion; it is also influenced by barriers (such as cell membranes), tissue organisation and microvascular perfusion, hence the term “apparent” [1,38]. It provides an objective measure of tissue cellularity and facilitates distinction between healthy and pathological tissue. The ADC represents the rate of diffusion-related signal attenuation as the b-value increases, reflecting the degree of water diffusion within tissue. If water moves freely, the signal drops quickly (high ADC). If water is restricted, the signal drops slowly (low ADC) (Figure 3).

The choice between the mean and minimum ADC involves a trade-off rather than a clear hierarchy. The minimum ADC has been reported to correlate more closely with histological grade in heterogeneous soft tissue tumours, on the reasoning that it samples the most cellular focus and is less diluted by necrotic, cystic or myxoid regions than a whole-lesion mean [12,42]. That advantage is not equivalent to robustness. Because it is an extreme value statistic, the minimum ADC is by construction sensitive to image noise, single outlying pixels, tissue-composition-related pitfalls, partial volume averaging at lesion margins, and subjective ROI placement, and it will tend to fall as ROI size or slice coverage increases. The schwannomas discussed in Section 4.5 illustrate the resulting hazard directly: focal Antoni A regions produce genuinely low minimum ADC values in a benign lesion, so that the metric most sensitive to hypercellular foci is also the metric most likely to mislead. Published reproducibility data for both metrics in musculoskeletal DWI are limited, and intraobserver and interobserver agreement for the minimum ADC is generally weaker than for the mean ADC. We therefore recommend that caution be exercised when using the minimum ADC value as a discriminator.

Region of interest (ROI) placement is critical for accurate and reproducible ADC measurement. The ROI should be placed in viable, non-necrotic, non-haemorrhagic tissue, avoiding artefacts, oedema, and adjacent normal structures [3]. Standardised reporting should document ROI dimensions, anatomical placement, and the specific ADC metric employed, i.e., whether the lowest, average, highest, or a normalised ratio value was used, as each carries distinct clinical implications. Expressing the ADC as a ratio relative to adjacent muscle, i.e., the lesion/muscle ADC ratio, reduces inter-scanner variability and improves cross-institutional comparability [3,8].

A general caution applies to every numerical ADC value and threshold quoted in this review. The ADC is not a scanner-independent physical constant of tissue. Reported values vary with field strength, vendor and gradient hardware, sequence design and echo time; the number and distribution of b-values and whether b = 0 is included; the fat-suppression method; the anatomical site and its susceptibility environment; slice thickness and partial volume effects; ROI size, placement and the metric extracted; and the lesion composition [3]. Although inter-scanner variability in ADC measurements has generally been reported in the low single-digit percentage range under harmonised acquisition conditions [43,44], substantially greater differences may occur when acquisition and analysis protocols vary. The values cited throughout this review are reproduced as published, together with their source, and should be read as indicative of direction and order of magnitude rather than as transferable cut-offs. Where a threshold was derived and tested within a single cohort, it should be regarded as hypothesis-generating until externally validated. Departments intending to use quantitative ADC clinically should establish their own reference ranges on their own equipment and protocol. Provenance of principle ADC thresholds is highlighted in Table 4. The provenance of the principal ADC thresholds cited in this review is summarised in Table 2.

### 2.4. Advanced Diffusion Sequences: DTI and DKI

DTI extends DWI by applying gradients in at least six non-collinear directions, enabling calculation of the full diffusion tensor and additional metrics, including FA and MD [5,6]. FA (ranging from 0 for isotropic to 1 for fully anisotropic diffusion) is particularly informative in highly organised structures such as tendons, ligaments, and peripheral nerves, where structural integrity correlates closely with anisotropy. Tractography based on DTI enables three-dimensional fibre tracking with important applications in presurgical nerve mapping [5,6].

Diffusion kurtosis imaging (DKI) examines non-Gaussian water diffusion behaviour, which standard DTI fails to capture in tissues with high structural complexity [7]. DKI calculates mean kurtosis (MK), axial kurtosis (AK), and radial kurtosis (RK), providing enhanced sensitivity to microstructural complexity in dense tissues such as cartilage and tumours. These metrics are derived using high b-values (>1000 s/mm^2^) and multi-directional gradients and offer complementary information beyond DTI for evaluating pathologies such as inflammation, oedema, and synovitis [7].

## 3. Normal MSK Tissue Appearances and the Composition–Signal Framework

On DW images, bones, muscles, and fatty tissues appear hypointense due to lack of significant mobile protons, rapid diffusion, or fat suppression. Arterial flow is completely suppressed, while slow-flow structures such as veins may be visible on low-b-value images due to T2 shine-through. Yellow bone marrow demonstrates low DWI signal and very low ADC values due to its predominant fat content, whereas red (cellular) marrow shows higher DWI signal and low ADC values, reflecting greater water content and cellularity [8,45]. This distinction is critical when differentiating normal red marrow from pathological infiltration. Reference ADC values for key MSK structures are summarised in Table 3.

### 3.1. The Composition–Signal Framework

The applications reviewed in Section 4, Section 5, Section 6, Section 7, Section 8, Section 9 and Section 10 rest on a single physical premise, and it is worth stating that premise explicitly before the applications are surveyed. The DWI signal is not a direct readout of cellularity. It is the joint product of three partly independent tissue properties: the mobility of water within the intracellular and extracellular compartments, the intrinsic T2 relaxation time of the tissue, and the density of mobile protons available to generate a signal at all. Conventional teaching collapses these three into a single expectation—that hypercellular tissue restricts water motion and therefore appears bright on high-b-value images with a correspondingly low ADC. Most musculoskeletal pathologies conform to this expectation. The cases that do not conform are conventionally catalogued as pitfalls and set aside.

We propose that these discordant cases are more usefully read as compositional evidence. Where the three determinants diverge, where a matrix holds abundant free water despite malignant cytology, where paramagnetic or crystalline material suppresses the signal irrespective of diffusion, or where viscous proteinaceous contents impede water motion in the near-absence of cells—the direction and magnitude of the discordance is itself specific to the underlying substrate. A malignant myxoid tumour that fails to restrict is not a failure of the sequence; it is the sequence reporting the presence of a myxoid matrix. A lesion dark on both DWI and the ADC map is not uninterpretable; it is reporting haemosiderin, mineral, or dense collagen. The interpretive task is therefore not to decide whether the signal is trustworthy but to identify which of the three determinants is dominating it.

Table 4 sets out this mapping for the substrates encountered in musculoskeletal practice, together with the expected DWI and ADC behaviour, the interpretive consequence, and the corresponding illustrative case. The framework is applied in two ways in what follows. In Section 4, Section 5, Section 6, Section 7, Section 8, Section 9 and Section 10, each established and emerging application is annotated where composition materially alters interpretation, with a forward reference to the relevant row. In Section 12, relating to composition-related interpretive pitfalls, the discordant cases are examined in detail, organised by substrate rather than by the name of the artefact, and illustrated by institutional cases with histological or surgical correlation.

### 3.2. Levels of Evidence for the Applications Reviewed

The applications surveyed in Section 4, Section 5, Section 6, Section 7, Section 8, Section 9 and Section 10 differ widely in the strength of the evidence supporting them, and grouping them under a single heading of clinical utility would misrepresent that difference. Whole-body DWI in multiple myeloma is supported by consensus reporting guidance and prospective multicentre data and is embedded in national imaging pathways. At the other extreme, ADC-based ageing of venous thrombus, diffusion metrics in inflammatory myopathy and peripheral neuropathy, DTI tractography of nerve sheath tumours, and paediatric surveillance protocols rest on small, single-centre or heterogeneous series whose thresholds have not been tested outside the cohorts that generated them. Table 5 makes this stratification explicit, and each application section states its tier at the outset. Throughout the manuscript, we have reserved the terms established, validated and superior for Tier 1 and, where the evidence specifically justifies it, Tier 2 applications.

## 4. Role of DWI in Soft Tissue Tumour Characterisation

**Evidence tier.** Tier 2 for benign versus malignant characterisation and for biopsy guidance; Tier 2 to 3 for treatment response; Tier 3 for postsurgical assessment and for peripheral nerve sheath tumour characterisation. Consistent evidence across independent cohorts supports the direction of the ADC findings, but ADC ranges overlap substantially between benign and malignant lesions, and no threshold has been validated for transfer between institutions (Table 5).

### 4.1. Benign Versus Malignant Differentiation

MSK tumours frequently demonstrate overlapping and non-specific appearances on conventional T1- and T2-weighted sequences, limiting diagnostic specificity [3,12]. DWI provides a quantitative complement to morphological MRI, with established utility in differentiating benign from malignant soft tissue masses, guiding high-yield regions for biopsy, and assessing treatment response [12,42,53].

**Figure 4:**
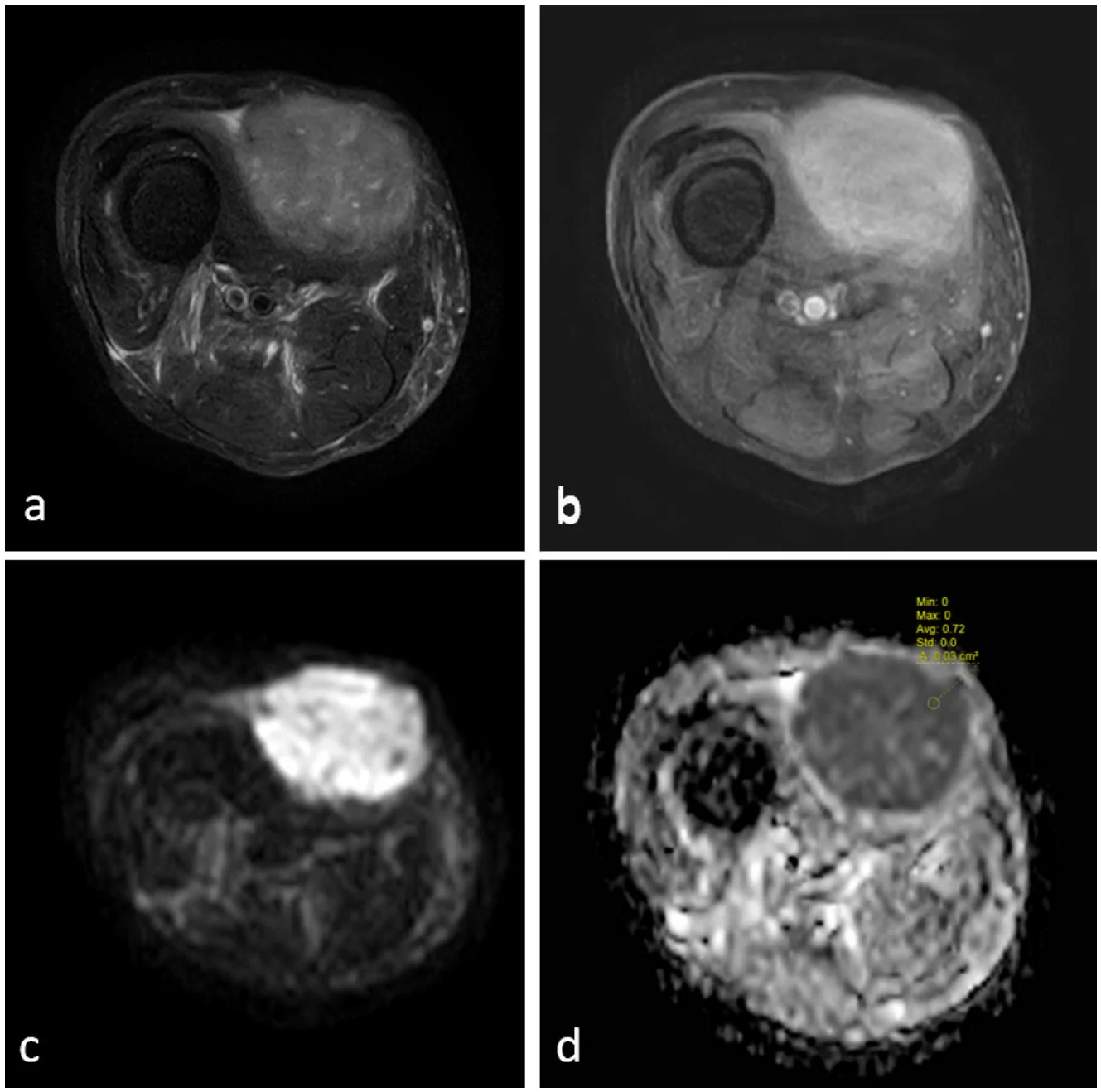
Axial MRI of a histopathologically confirmed soft tissue sarcoma demonstrating a fat-suppressed T2-weighted hypointense mass (**a**) with homogeneous post-contrast enhancement (**b**), restricted diffusion on DWI (**c**), and correspondingly low signal on the ADC map (**d**). Quantitative ADC measurement yielded a value of 0.7 × 10^−3^ mm^2^/s, consistent with the reported range for malignant soft tissue tumours.

The biological basis for DWI’s diagnostic utility lies in the relationship between cellularity and water diffusion. Malignant tumours tend to have high cellular density, with tightly packed cells and abundant intact cell membranes that restrict the movement of water molecules. This results in restricted diffusion, characterised by high signal intensity on diffusion-weighted imaging (DWI) and low signal intensity on apparent diffusion coefficient (ADC) maps (Figure 4) [3,54].

Benign lesions tend to be hypocellular, allowing relatively free water diffusion. Free diffusion is qualitatively visible as progressive signal loss on DWI images obtained with increasing b-values (as perfusion effects diminish) and quantitatively as high ADC values, indicating hypocellularity and suggesting benignity [3].

A minimum ADC value below 1.0 × 10^−3^ mm^2^/s is more frequently observed in malignant soft tissue tumours and is considered a stronger predictor of malignancy than mean ADC [42,55]. Razek et al. reported a mean ADC cut-off of 1.34 × 10^−3^ mm^2^/s, achieving accuracy of 91%, sensitivity of 94%, and specificity of 88% for excluding malignancy [9]. Subhawong et al. demonstrated that a mean ADC > 1.7 and a minimum ADC > 1.3 × 10^−3^ mm^2^/s yielded sensitivity of 40% and specificity of 92% for excluding malignant soft tissue masses [10]. Del Grande et al. identified minimum ADC ≤ 0.8 × 10^−3^ mm^2^/s as the only significant independent predictor of malignancy, with sensitivity of 75% and specificity of 73% [11].

**Figure 5:**
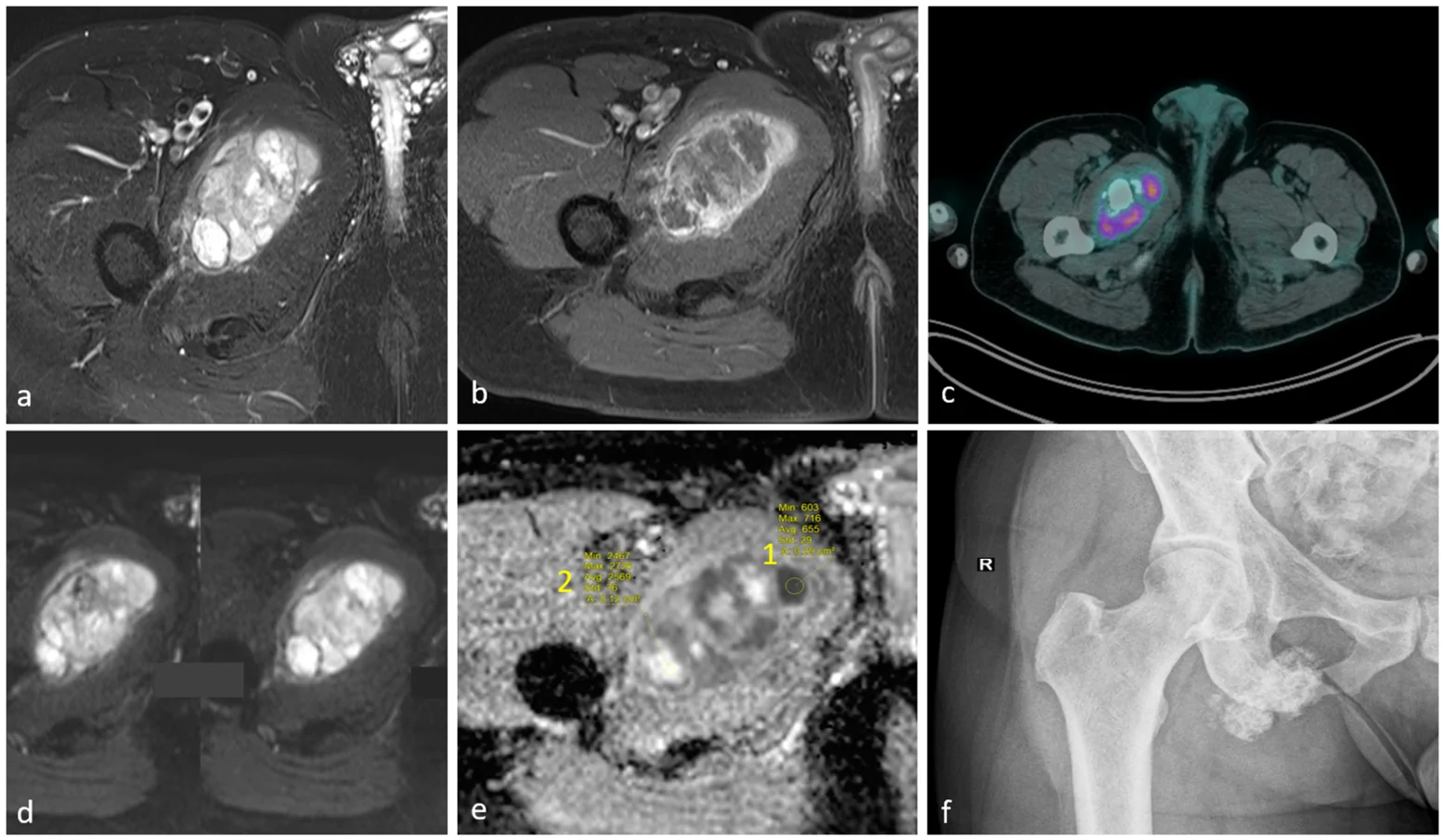
Axial MRI, PET-CT, and radiograph of a biopsy-proven extraskeletal mesenchymal chondrosarcoma. Fat-suppressed T2-weighted imaging (**a**) demonstrates a heterogeneous mass with mixed signal intensity. Post-contrast T1-weighted imaging (**b**) reveals peripheral enhancement with a central peripheral and septal chondroid pattern of enhancement. DWI (**d**) shows heterogeneous bright signal, with the ADC map (**e**) identifying an eccentric focus of true restricted diffusion corresponding to the round cell sarcomatous component (1) and a separate focus of T2 shine-through representing the chondroid component (2). FDG PET-CT (**c**) confirms peripheral metabolic activity with central calcification, and the frontal radiograph of the right hip (**f**) demonstrates characteristic ring-and-arc calcifications. This case highlights the role of DWI in characterising tumour heterogeneity—the sarcomatous component exhibits restricted diffusion and avid enhancement, whilst the chondroid component is responsible for T2 shine-through and the characteristic calcification pattern.

**Figure 6:**
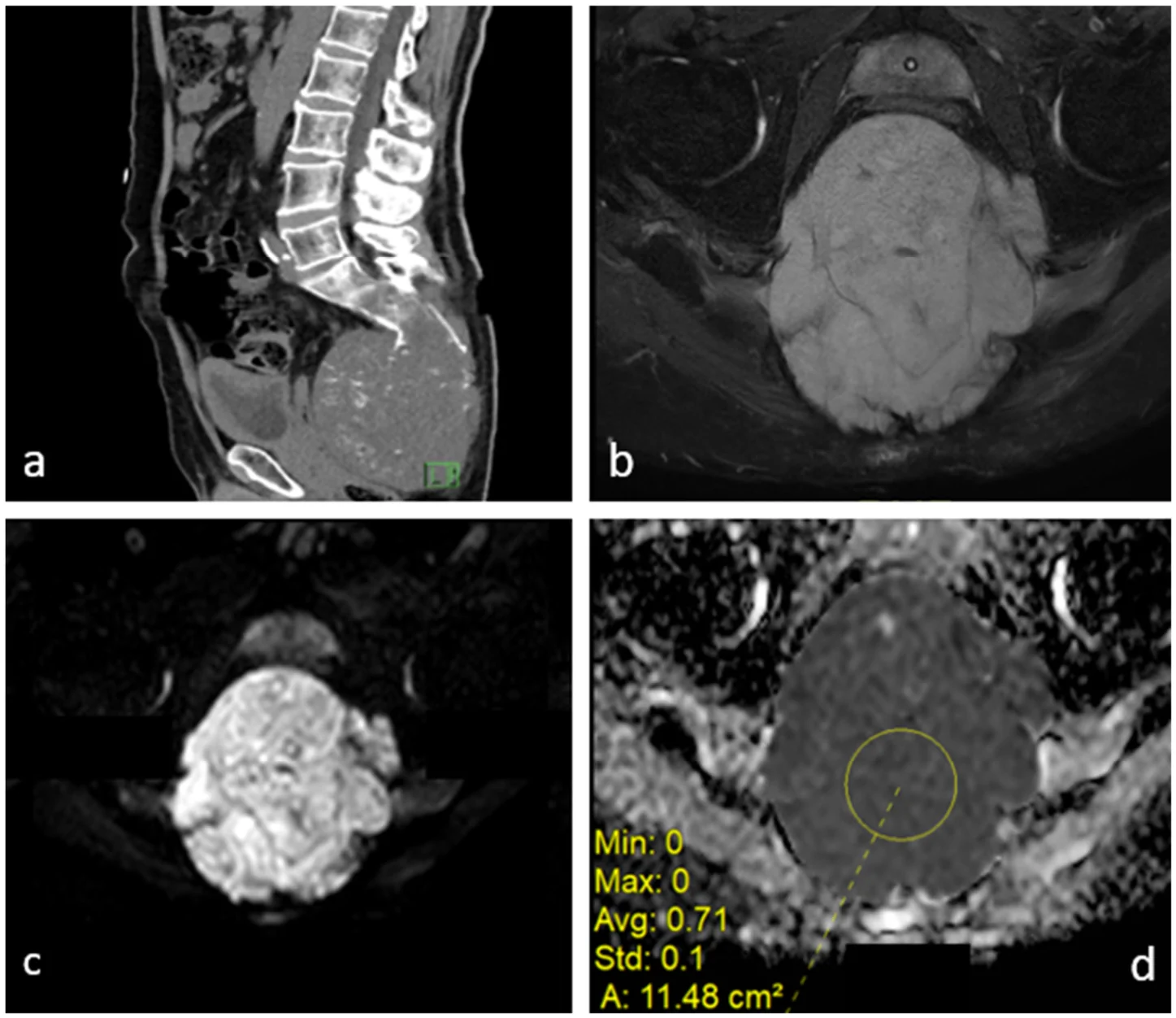
Diffusion-weighted imaging DWI is useful in distinguishing chordoma (histologically proven) from chondrosarcoma. The two entities can be difficult to differentiate on CT (**a**), and both typically demonstrate high signal intensity on T2-weighted imaging (**b**). On diffusion-weighted imaging (**c**), however, chondrosarcoma is characteristically associated with higher apparent diffusion coefficient (ADC) values, whereas chordoma shows restricted diffusion with correspondingly low ADC values (**d**) [56].

However, several compositional factors limit the reliability of absolute ADC thresholds. Fibrous tumours such as mature desmoid fibromatosis exhibit low ADC values due to the T2 black-out effect, potentially mimicking malignancy. Conversely, myxoid tumours, chondroid lesions, and cystic masses demonstrate high ADC values despite possible malignancy (Figure 5). Foci of mineralisation, haemorrhage, cortical bone, and macroscopic fat all produce erroneously low ADC values through technical rather than biological effects (Figure 6) [3,12,54]. A multiparametric approach integrating DWI with clinical history and conventional MRI sequences is therefore essential [3,8].

These compositional effects are not incidental limitations but the systematic behaviour set out in Table 4. Fibrous and desmoid-type lesions (Table 4, row 3) and hemosiderin-laden lesions (row 2) yield spuriously low ADC values that may be read as malignancy, whereas myxoid and chondroid matrices (rows 1 and 11) yield high ADC values that may be read as benignity in a histologically malignant lesion. Each is examined in detail, with illustrative institutional cases, in Section 12.

### 4.2. Biopsy Guidance

In heterogeneous tumours, ADC mapping identifies the most hypercellular regions—areas of high DWI signal and low ADC—for targeted percutaneous biopsy, maximising diagnostic yield and reducing sampling of necrotic or cystic areas [3,8]. This is particularly relevant in large or heterogeneously enhancing masses where conventional MRI alone cannot reliably delineate viable tumours from treatment effects or necrosis. Multiparametric bone MRI incorporating DWI has been shown to improve CT-guided biopsy target selection and increase diagnostic yield in cancer patients [37].

### 4.3. Treatment Response Following Radiotherapy/Chemotherapy

Conventional post-treatment assessment relies on anatomical MRI, where response is inferred from reductions in lesion size and contrast enhancement. However, these parameters can be unreliable, as treatment-related fibrosis and granulation tissue often demonstrate persistent or even increased enhancement, thereby mimicking residual viable tumours [13,14,15].

Diffusion-weighted imaging (DWI), through apparent diffusion coefficient (ADC) quantification, provides a valuable adjunct by enabling differentiation between viable and non-viable tissue. Several studies support its utility. Einarsdóttir et al. demonstrated that post-radiation increases in ADC values correlated with 40–60% tumour necrosis [13]. Dudeck et al. reported an inverse relationship between ADC values and tumour volume following treatment, reinforcing the ADC as a marker of therapeutic response [14]. Similarly, Soldatos et al. found that inflammatory post-treatment tissue, while enhancing on contrast imaging, exhibited higher ADC values consistent with favourable histologic response. They proposed a minimum ADC threshold of >2.0 × 10^−3^ mm^2^/s as indicative of good response, with high sensitivity (100%) but moderate specificity (61%) [15].

### 4.4. Postsurgical Assessment of Residual or Recurrent Disease

In the postoperative setting, conventional MRI is limited by the presence of enhancing scar tissue, which can mimic residual or recurrent tumours. DWI improves diagnostic performance in this context. Del Grande et al. showed that the presence of a hypointense mass on ADC maps significantly increased specificity for detecting residual or recurrent disease from 52% to 97%, albeit with a sensitivity of 60% [11]. In studies of unplanned soft tissue sarcoma resections, residual tumours consistently demonstrated lower ADC values compared to surrounding vasogenic oedema, with minimum ADC values around 1.16 × 10^−3^ mm^2^/s (range: 0.58–1.59 × 10^−3^ mm^2^/s).

The integration of DWI and ADC mapping into routine MRI protocols substantially enhances diagnostic accuracy in both post-treatment response evaluation and postsurgical surveillance. By providing quantitative insight into tissue cellularity and viability, DWI addresses key limitations of conventional imaging and supports more precise clinical decision-making.

### 4.5. Peripheral Nerve Sheath Tumour Characterisation and DTI Tractography

DWI may play an important role in the characterisation of peripheral nerve sheath tumours (PNSTs), particularly in distinguishing benign from malignant entities.

Benign PNSTs such as schwannomas and neurofibromas typically demonstrate higher mean and minimum ADC values, reflecting relatively less restricted diffusion. In contrast, malignant peripheral nerve sheath tumours (MPNSTs) show lower mean and minimum ADC values, corresponding to increased cellularity and more pronounced diffusion restriction.

**Figure 7:**
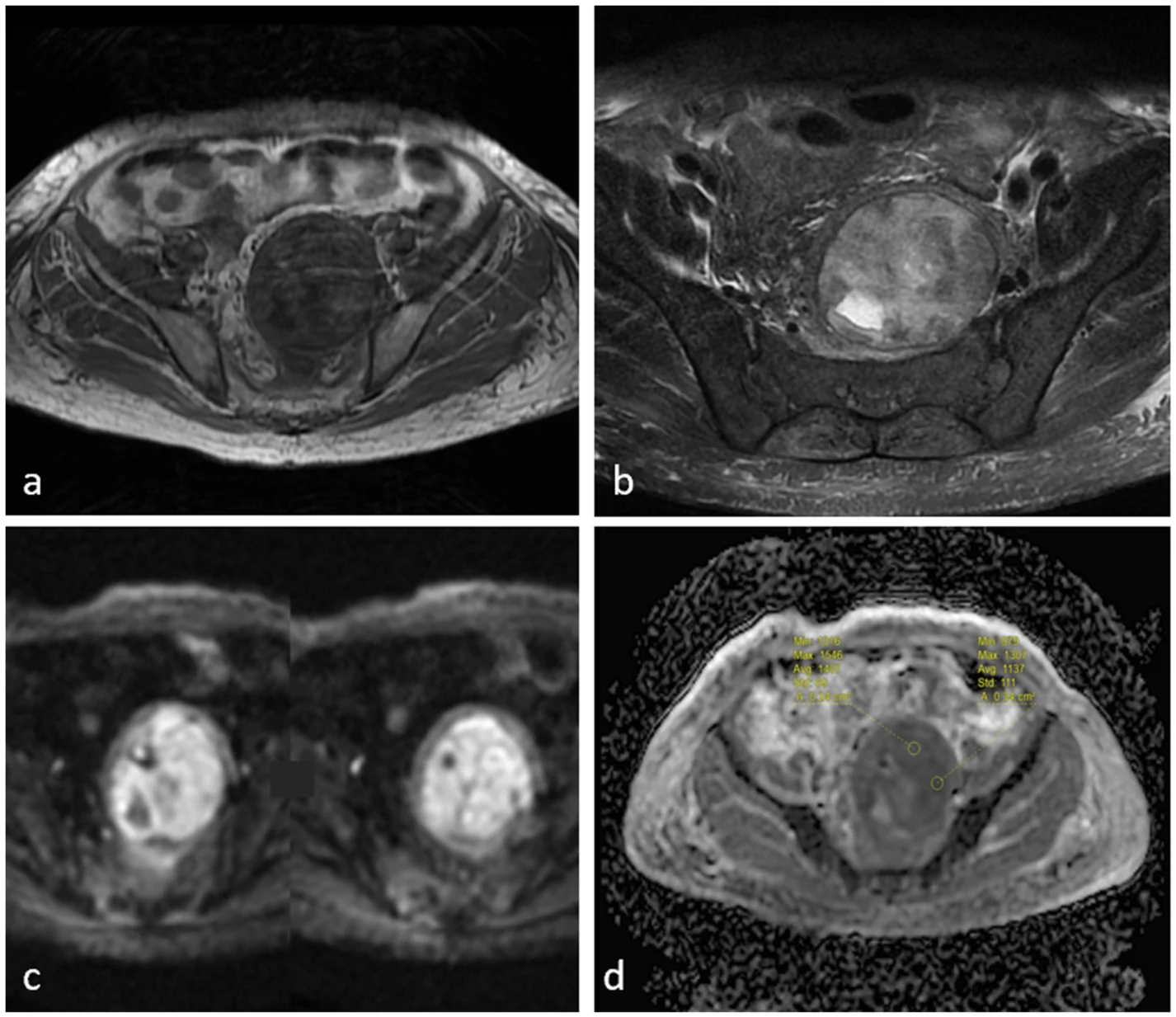
Axial MRI of a pelvic schwannoma (histopathology proven) (hi): T1-weighted hypointense (**a**) and heterogeneously T2-weighted hyperintense (**b**), with high signal on DWI (**c**). Despite heterogeneous areas of high and low ADC (**d**) values (due to Antoni A and Antoni B regions), quantitative measurement yielded a mean ADC value of 1.5 × 10^−3^ mm^2^/s, excluding true restriction. This case underscores the importance of quantitative over qualitative ADC assessment in peripheral nerve sheath tumours; an ADC below 1.0 × 10^−3^ mm^2^/s should raise suspicion for malignant transformation (MPNST), while higher values favour a benign aetiology.

However, important diagnostic pitfalls exist. Schwannomas, for example (Figure 7), exhibit a heterogeneous histological architecture composed of Antoni A regions (densely packed spindle cells) and Antoni B regions (loosely arranged, myxoid, hypocellular tissue). This results in mixed diffusion characteristics, with areas of restricted diffusion (low ADC) corresponding to Antoni A regions and facilitated diffusion (higher ADC) in Antoni B regions. Consequently, reliance on focal minimum ADC values may be misleading. In such cases, the average ADC value provides a more representative assessment and supports a benign diagnosis. Correlation with conventional MRI sequences remains essential.

**Figure 8:**
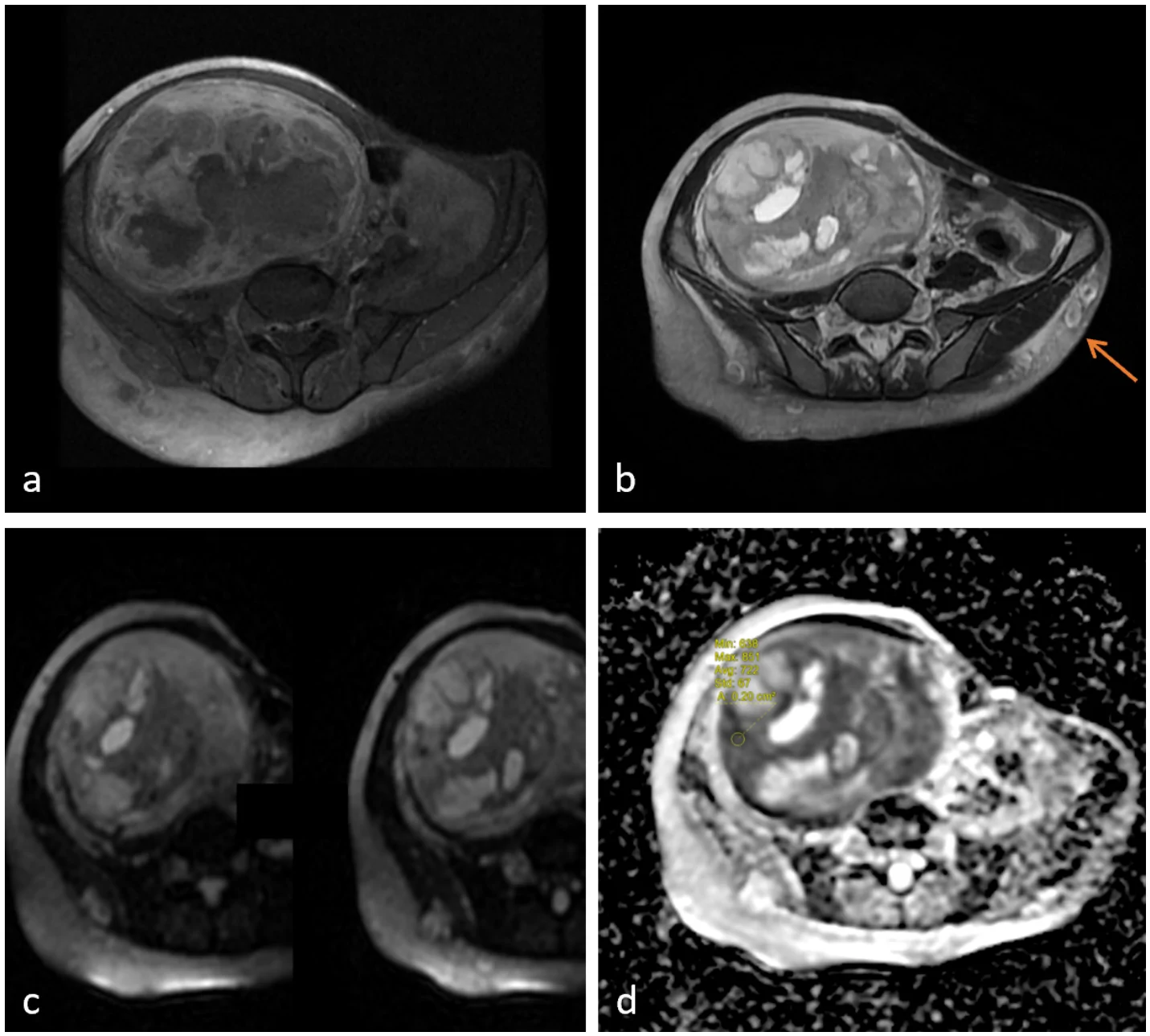
Axial MRI in a patient with neurofibromatosis type 1 demonstrating a large heterogeneous pelvic mass on T1-weighted (**a**) and T2-weighted (**b**) imaging. DWI (**c**) reveals heterogeneous signal with multifocal areas of significant restricted diffusion and markedly low ADC (**d**) values (<1.0 × 10^−3^ mm^2^/s), consistent with a malignant peripheral nerve sheath tumour (MPNST) (histopathology-proven). Incidental small benign neurofibromas are noted in the left gluteal region (orange arrow).

Differentiation of benign from malignant PNSTs is also clinically important in patients with neurofibromatosis type 1 (NF1), where patients carry an approximately 10% lifetime risk of malignant transformation [27] (Figure 8). Benign PNSTs demonstrate higher mean and minimum ADC values, while MPNSTs show more restricted diffusion; a minimum ADC ≤ 1.0 × 10^−3^ mm^2^/s has been proposed as a strong indicator of malignancy, achieving 100% sensitivity and negative predictive value in selected series [27,28]. Additionally, tumour size greater than 4.2 cm further supported the diagnosis of malignancy [29]. Whole-body MRI with DWI is increasingly employed in NF1 surveillance to efficiently assess tumour burden and detect malignant transformation [27].

Furthermore, DTI with tractography provides unique information about the spatial relationship between nerve sheath tumours and adjacent nerve fibres, supporting precise presurgical planning [5,57]. Schwannomas typically displace nerve fibres around the tumour; neurofibromas show splayed fibres traversing through the lesion; and malignant peripheral nerve sheath tumours (MPNSTs) demonstrate infiltrated and disrupted fibre tracts [57]. This characterisation guides nerve-sparing surgical strategies, though clinical use remains constrained by long acquisition times, susceptibility to artefacts at fibre crossings and interpretive complexity [57]. In summary, while DWI is a valuable adjunct in PNST evaluation, accurate interpretation requires careful quantitative ADC analysis and integration with morphological imaging findings to avoid misclassification. The characteristic tractography fibre patterns and their ADC correlates are summarised in Table 6.

## 5. DWI of Bone Marrow Lesions

**Evidence tier.** Tier 1 for myeloma staging and response assessment under MY-RADS, supported by consensus guidance and prospective multicentre data; Tier 2 for skeletal metastasis assessment in solid tumours; Tier 2 for lesion characterisation and for discrimination of benign from malignant vertebral compression fractures. Tier 2 for characterisation of focal marrow lesions when combined with Dixon fat-fraction and T1-weighted correlation. Quantitative marrow ADC ranges differ markedly between published sources and acquisition settings and should not be used as absolute thresholds (Section 2.3, Table 5). Tier 2 to 3 for treatment response monitoring, where cut-offs derive from small cohorts (Table 5).

### 5.1. Whole-Body DWI: Myeloma Staging and Response Assessment

**Figure 9:**
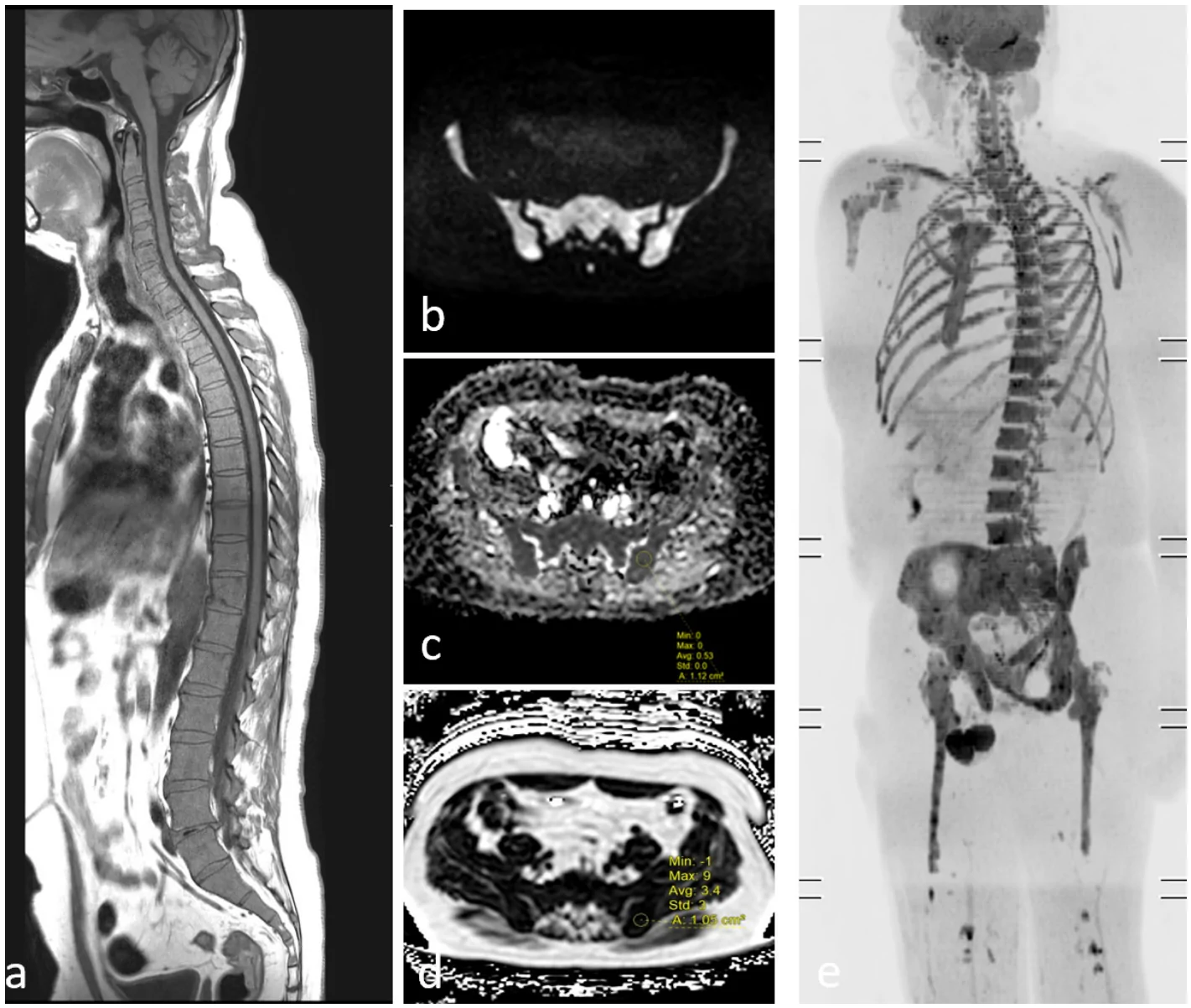
Whole-body MRI in a patient with multiple myeloma (histopathology-proven) demonstrating diffuse T1-weighted hypointense marrow on the sagittal image (**a**), with corresponding diffuse high signal on axial DWI (**b**) and restricted diffusion on the ADC map (**c**), consistent with hypercellular marrow infiltration. Replacement of normal marrow fat is confirmed on fat fraction sequences (**d**). The inverted MIP reconstruction of the DWI dataset (**e**) provides a comprehensive whole-body overview of all sites of high diffusion signal, analogous to a PET maximum intensity projection.

**Figure 10:**
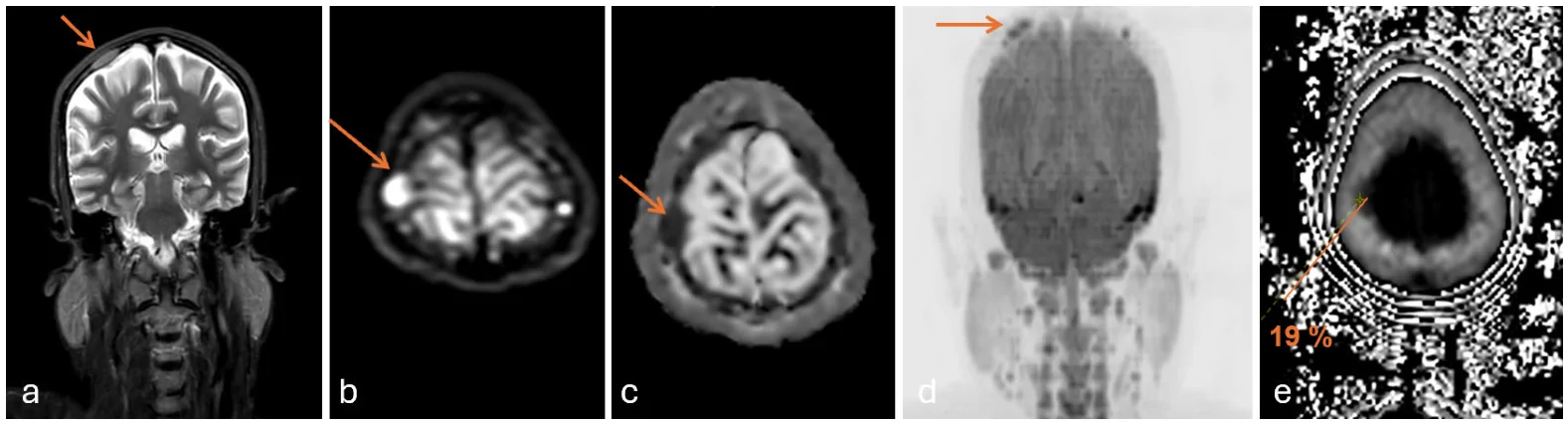
Whole-body MRI demonstrating an isolated myeloma (histopathology proven) deposit in the right parietal skull (orange arrows). Coronal STIR imaging (**a**) reveals a STIR hyperintense lesion, confirmed on DWI (**b**) with corresponding restricted diffusion on the ADC map (**c**). The inverted MIP reconstruction (**d**) provides improved conspicuity of the lesion against the suppressed background. Fat fraction imaging (**e**) demonstrates marrow replacement at the lesion site, with a fat fraction below 20% considered significant for pathological marrow infiltration.

WB-MRI with DWI is validated within the MY-RADS framework for myeloma staging and treatment response assessment, offering superior sensitivity over conventional skeletal scintigraphy for detecting diffuse and focal marrow disease [18,58]. At our institution, the MR skeletal survey protocol for multiple myeloma comprises sagittal T1 of the spine, large-field-of-view coronal T1 and STIR sequences covering the vertex to knee, and axial DWI (b = 0 and 1000 s/mm^2^) with ADC and relative fat fraction (rFF) maps over the same coverage. This protocol is employed for myeloma staging and skeletal metastasis assessment [18,59] (Figure 9 and Figure 10).

In multiple myeloma, baseline ADC values have been proposed as independent prognostic markers for disease progression and death [18]. Whole-body MRI incorporating DWI is validated for myeloma staging and response assessment within the MY-RADS framework. ADC values serve as quantitative response markers, with rising ADC reflecting treatment-related cell death and reduced tumour burden [18,58]. WB-DWI with MY-RADS is now the recommended standard for myeloma staging in the UK. DWI sensitivity for focal lesion detection is 77% versus 47% for FDG-PET/CT, and spine-only imaging misses 50% of lesions [19]. The superiority of DWI over conventional morphological MRI for response assessment is well established: Giles et al. demonstrated ADC rise in 95% of responders versus decline in all non-responders [20]. In contrast, the IMAJEM trial showed conventional MRI normalised in only 3–11% of patients during treatment with no predictive value for PFS or OS, while the authors explicitly proposed DWI-MRI as the superior functional alternative [21].

MY-RADS defines ADC thresholds: normal marrow < 600–700 µm^2^/s, viable tumour 700–1400 µm^2^/s, and treated/necrotic tissue ≥ 1400 µm^2^/s [19]. Responding lesions show an early ADC rise at 4–6 weeks followed by a decline as fatty marrow reconstitutes [19,20]. The five-point RAC scoring system (RAC 1 = highly likely responding; RAC 5 = highly likely progressing) integrates ADC changes, high-b-value signal, and morphology. False positives include fractures, infection, haemangiomas, implant artefacts, and rebound marrow hyperplasia, which require systematic exclusion by Dixon fat fraction and T1 correlation [19].

The prospective iTIMM trial confirmed that MY-RADS WB-MRI provides prognostic information independent of serology and bone marrow MRD (minimal residual disease) and reflects the complementary and non-redundant roles of imaging and laboratory MRD assessment [22].

**Figure 11:**
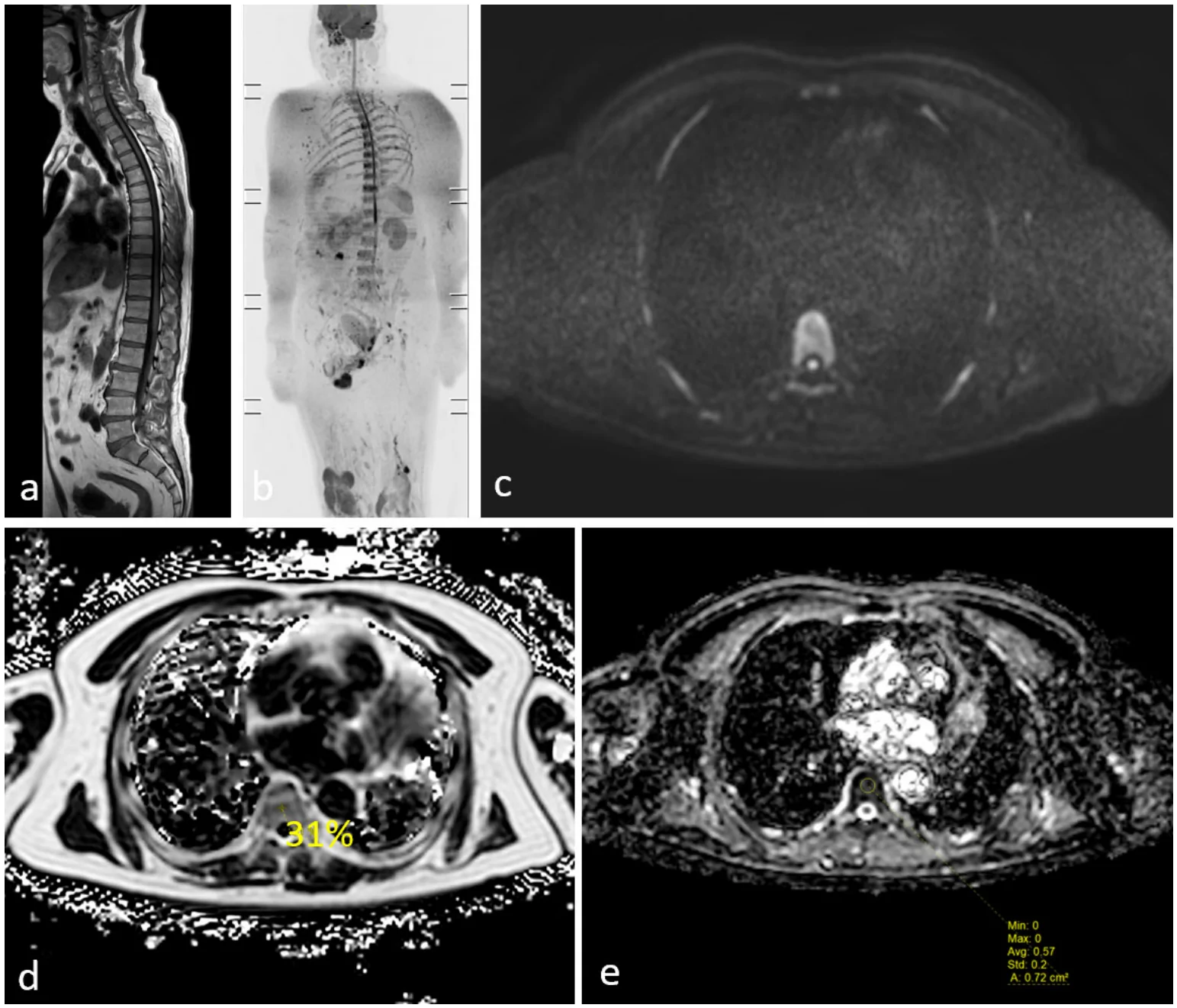
Whole-body MRI illustrating a recognised pitfall of diffusion-weighted imaging (image-based diagnosis only). Sagittal T1-weighted imaging (**a**) demonstrates preserved fatty marrow signal throughout the spine, and the inverted MIP reconstruction (**b**) highlights focal areas of high DWI signal in several thoracic vertebrae (**c**) with apparent restricted diffusion on the ADC map (**e**). However, fat fraction imaging (**d**) confirms normal marrow fat content, with no evidence of pathological infiltration—a fat fraction below 20% being the accepted threshold for significant marrow replacement. This case represents cellular hyperactive hematopoietic red marrow (30–60% fat fraction), which may be seen in chronic anaemia, smokers, etc., an important and potentially misleading pitfall of DWI interpretation that may falsely suggest myelomatous infiltration in the absence of true pathology.

Distinguishing marrow infiltration in diffuse myeloma from red marrow hyperplasia is aided by combined DWI and Dixon imaging assessment (Figure 11) [3,60].

### 5.2. Skeletal Metastases

Beyond myeloma, WB-DWI is validated as a one-step staging tool for skeletal metastases in solid tumours, particularly prostate cancer [61]. The key advantage over scintigraphy is direct detection of marrow infiltration rather than osteoblastic reaction. ADC quantification provides an early non-invasive treatment response biomarker where a rising ADC reflects tumour cell death and a falling ADC signals progression [61]. Standardised frameworks (MET-RADS-P for prostate cancer; MY-RADS for myeloma) provide structured five-point response assessments that integrate ADC changes with morphology [19,61].

In high-grade bone sarcomas, WB-MRI with DWI is under investigation for detecting bone marrow metastases [62].

### 5.3. DWI for Assessment of Vertebral Lesions and Compression Fractures

Assessment of bone marrow presents unique challenges for DWI due to competing signals from red marrow, yellow marrow, fat, and haematopoietic elements [45]. Red marrow has a higher ADC (approximately 0.90–1.34 × 10^−3^ mm^2^/s) compared to yellow marrow (0.36–0.50 × 10^−3^ mm^2^/s), reflecting differences in water content and cellularity [45,63]. These figures are higher than the indicative ranges given in Table 3, which are drawn from a different source and acquisition setting; the discrepancy illustrates the platform and protocol dependence discussed in Section 2.3 rather than a disagreement about marrow physiology, and neither range should be applied as a threshold outside the conditions in which it was measured. Distinguishing physiological red marrow reconversion from pathological infiltration is a key clinical challenge; chemical shift (Dixon) imaging is frequently combined with DWI to improve specificity, with a signal drop of > 50% on opposed-phase imaging indicating benign marrow with fat and water [3].

**Figure 12:**
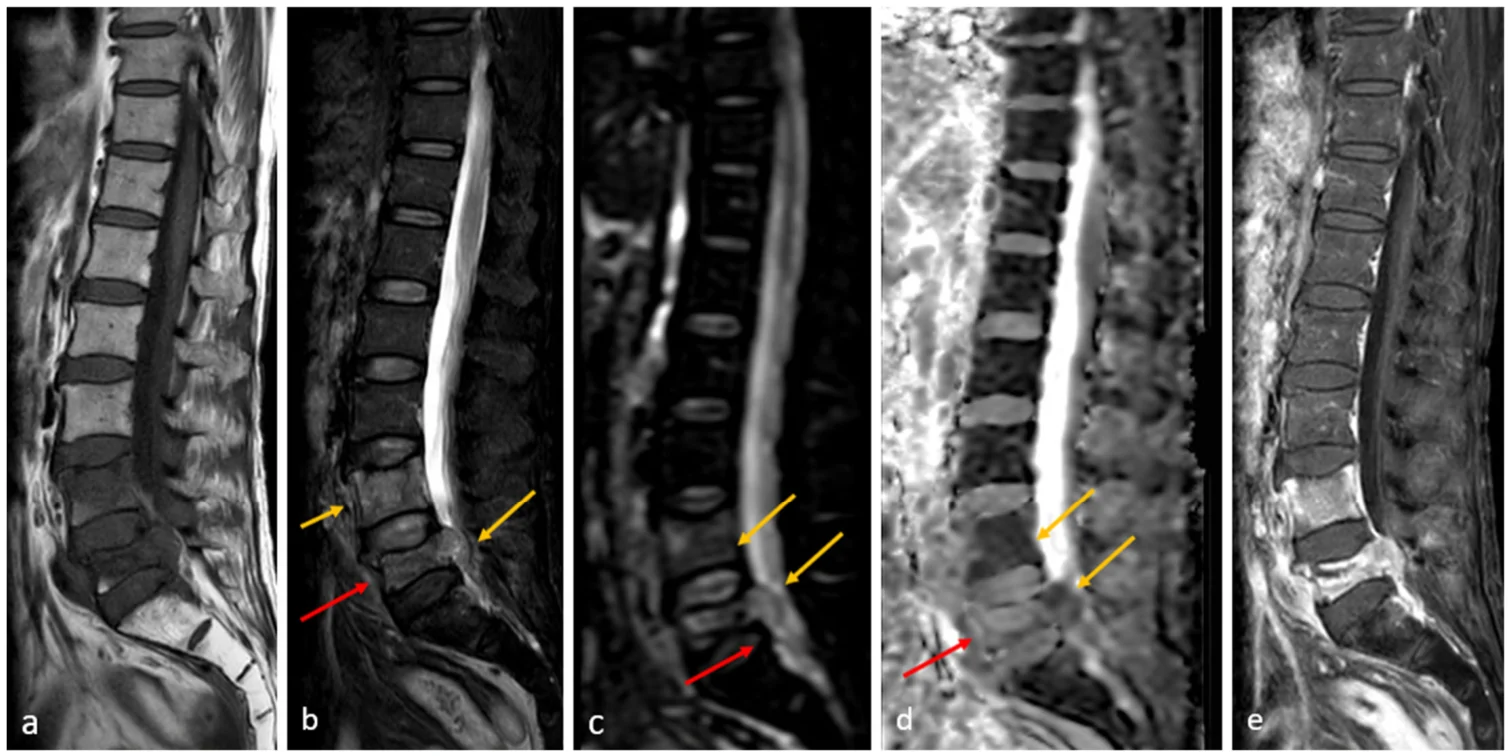
Diffusion-weighted imaging in the differentiation of benign from pathological vertebral compression fractures (imaging appearance in appropriate clinical context). The L4 vertebra and the posterior cortex of the L5 vertebral body (yellow arrows) demonstrate true restricted diffusion—hyperintense on T2-weighted (**b**) and DWI (**c**) images with corresponding hypointensity on the ADC map (**d**)—consistent with metastatic infiltration. In contrast, the L5 vertebral body, which shows the compression fracture, exhibits T2 shine-through (red arrows): hyperintensity on T2-weighted, DWI, and ADC images, attributable to vasogenic oedema. All these lesions appear hypointense on T1 (**a**), and post-contrast imaging (**e**) shows similar enhancement in both the metastatic lesions and the region of vasogenic oedema and is therefore of limited value in distinguishing the two.

In vertebral lesion characterisation, DWI differentiates metastatic infiltration from benign entities such as atypical haemangiomas, with metastases demonstrating significantly lower ADC values (Figure 12) [64]. In their meta-analysis, Suh et al. reported that the ADC demonstrated pooled sensitivity and specificity of 89% and 87%, respectively, for differentiating benign from malignant vertebral bone marrow lesions. For compression fractures, the corresponding sensitivity and specificity were 92% and 91%. Meta-regression identified DWI slice thickness as a significant source of heterogeneity (*p* < 0.01), with thinner slices (<5 mm) achieving higher specificity than thicker slices (95% vs. 81%) [16].

### 5.4. Role in Assessment of Focal Bone Lesions and Pathological Fractures

**Figure 13:**
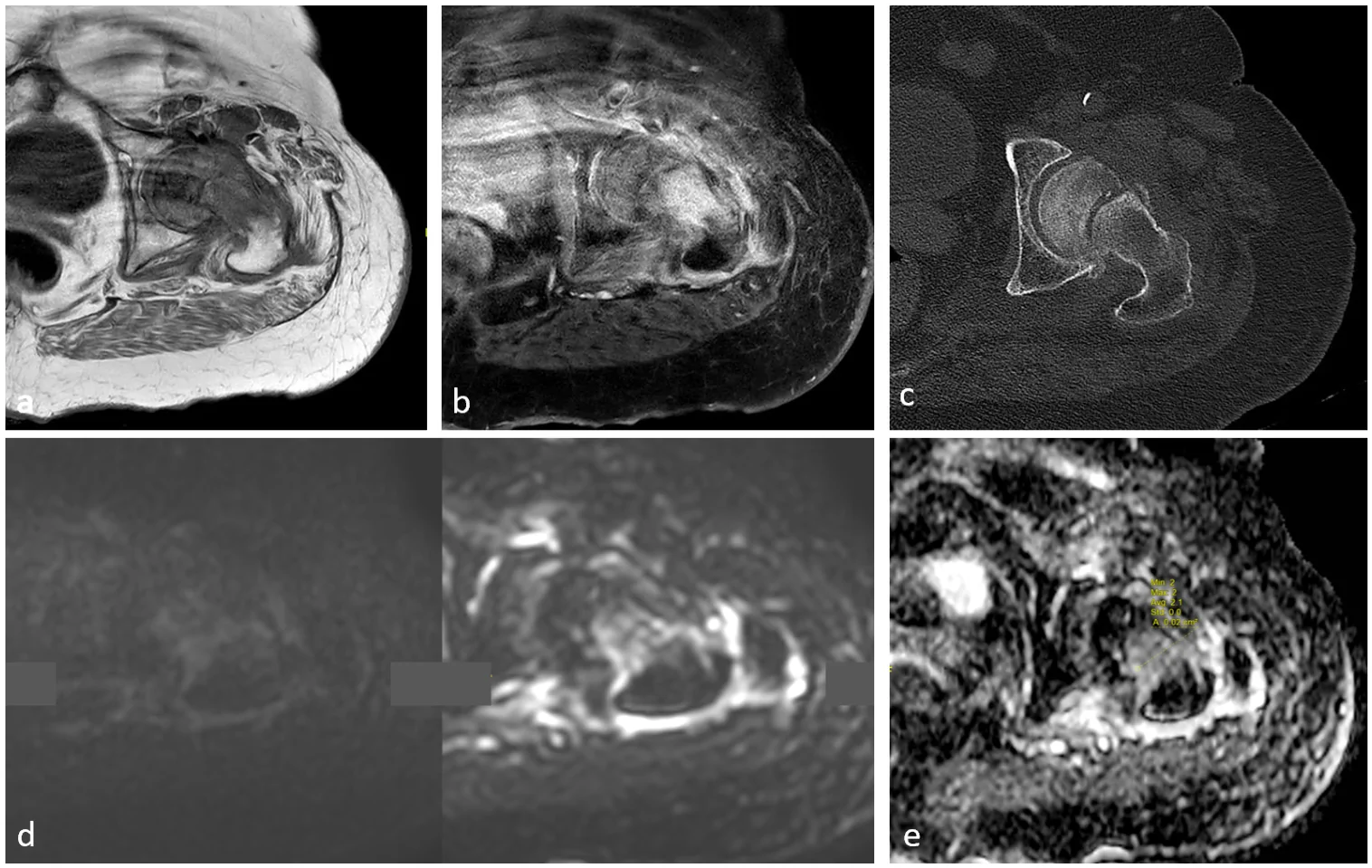
Axial MRI and CT of an elderly patient with trivial trauma, performed to exclude a pathological fracture of the femoral neck. Reactive vasogenic oedema surrounding the fracture site demonstrates T1-weighted hypointensity (**a**) with high signal on both DWI and the ADC map (**d**,**e**), reflecting T2 shine-through rather than true restricted diffusion. The absence of focal restricted diffusion supports a benign fracture when taken together with the morphology, clinical history and surgical fixation; some malignant lesions may show little or no restriction. Notably, significant reactive post-contrast enhancement is present at the fracture site (**b**), which may obscure or mimic pathological enhancement from an underlying lesion. CT (**c**) showed fracture without underlying lytic lesion.

**Figure 14:**
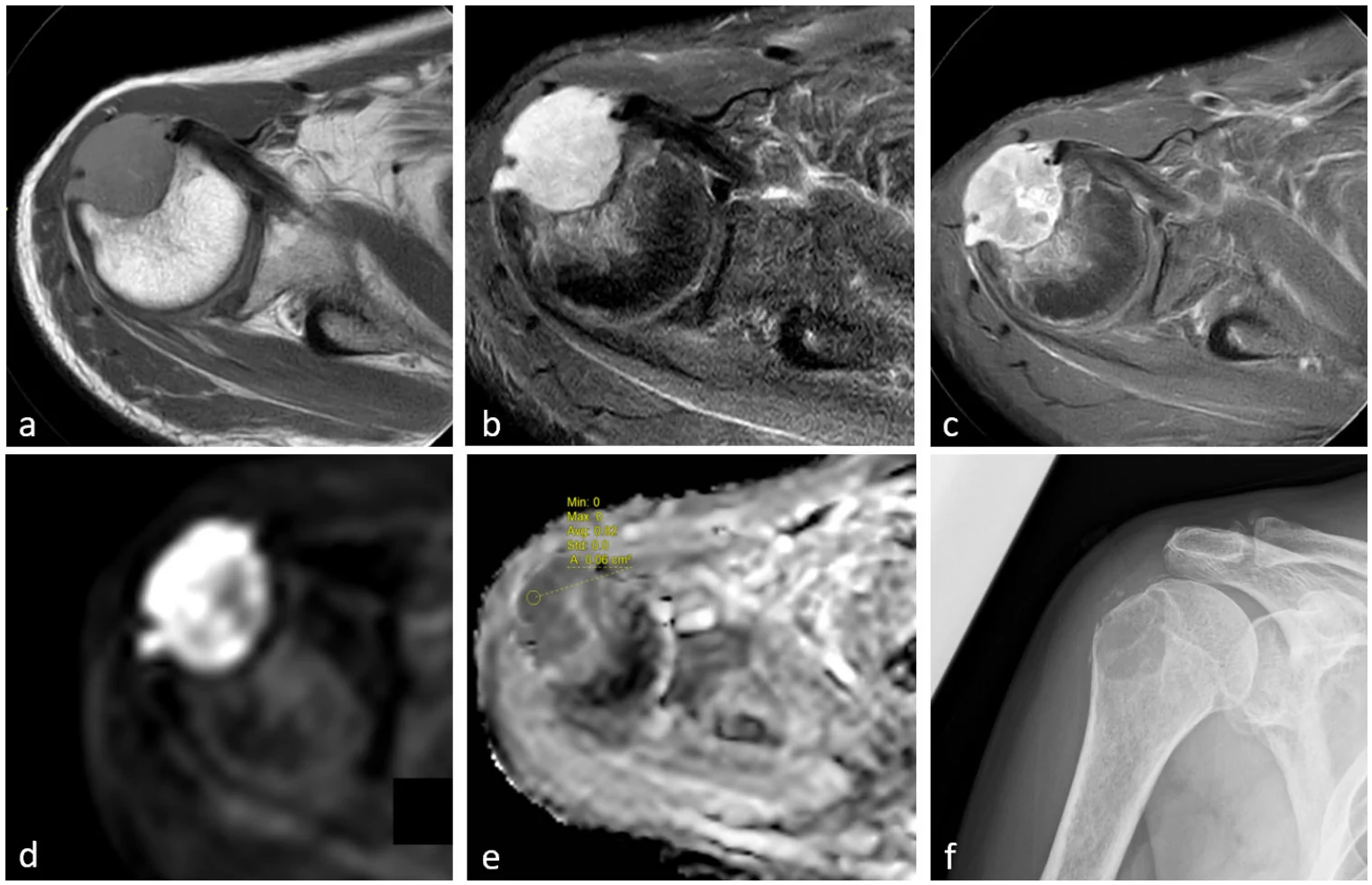
Axial MRI and radiograph demonstrating a metastatic lesion in the humeral head (image-based diagnosis only). The lesion is T1-weighted hypointense (**a**) and fat-suppressed T2-weighted hyperintense (**b**) and demonstrates post-contrast enhancement (**c**), with restricted diffusion on DWI (**d**) and the ADC map (**e**). The frontal radiograph (**f**) confirms a corresponding lytic lesion.

DWI significantly enhances conventional MRI evaluation of bone tumours by improving lesion conspicuity through background suppression and providing quantitative data on tumour cellularity [3,8]. Benign bone lesions, including bone cysts, ganglia, and vascular malformations, typically exhibit the T2 shine-through effect, appearing bright on DWI with correspondingly high ADC values reflecting facilitated rather than true restricted diffusion (Figure 13) [3,8]. Malignant bone lesions, particularly metastases, high-grade neoplasms and round cell tumours, such as lymphoma and myeloma, demonstrate true restricted diffusion with low ADC values (Figure 14). An ADC cut-off above 1.15 × 10^−3^ mm^2^/s has been proposed as indicative of a benign lesion, though notable exceptions exist [3].

DWI is less effective for tumours with chondroid or myxoid components. Chondrosarcoma and tumours with prominent myxoid stroma often do not demonstrate ADC restriction despite their malignant nature. This is because these tumours are characterised by an abundant extracellular matrix rich in glycosaminoglycans, which bind large amounts of water, resulting in a loose, highly hydrated matrix with relatively low cellular density, and are therefore not reliably characterised by DWI alone [3,8]. In the vertebral column, DWI differentiates malignant from benign osteoporotic compression fractures with pooled sensitivities and specificities of 89–92% [16].

### 5.5. Treatment Response Monitoring in Bone Sarcomas

Histologic tumour necrosis ≥ 90–95% following neoadjuvant chemotherapy is a critical prognostic marker in bone sarcomas, correlating with lower local recurrence rates and improved 5–10-year survival [17]. Conventional MRI-based assessment is challenged by tumour enlargement from inflammatory changes, haemorrhage, or necrosis, despite significant tumour kill. DWI addresses this limitation: successful treatment is reflected by rising mean ADC values, as cellular necrosis increases extracellular water mobility [17]. Significant differences in ADC between treatment responders and non-responders have been demonstrated across multiple studies, even when tumour volumes remain similar on conventional imaging [17]. ADC changes therefore represent a functionally sensitive and potentially contrast-free surrogate for histological necrosis in high-grade bone sarcomas.

## 6. DWI in Musculoskeletal Infections

**Evidence tier.** Tier 2 for detection and delineation of soft tissue abscesses, particularly where contrast is contraindicated; Tier 3 for the ADC ghost sign in osteomyelitis, which is highly specific but has a reported sensitivity of only approximately 20% and derives from a single retrospective series (Table 5).

### 6.1. Abscess Detection and Soft Tissue Infection

**Figure 15:**
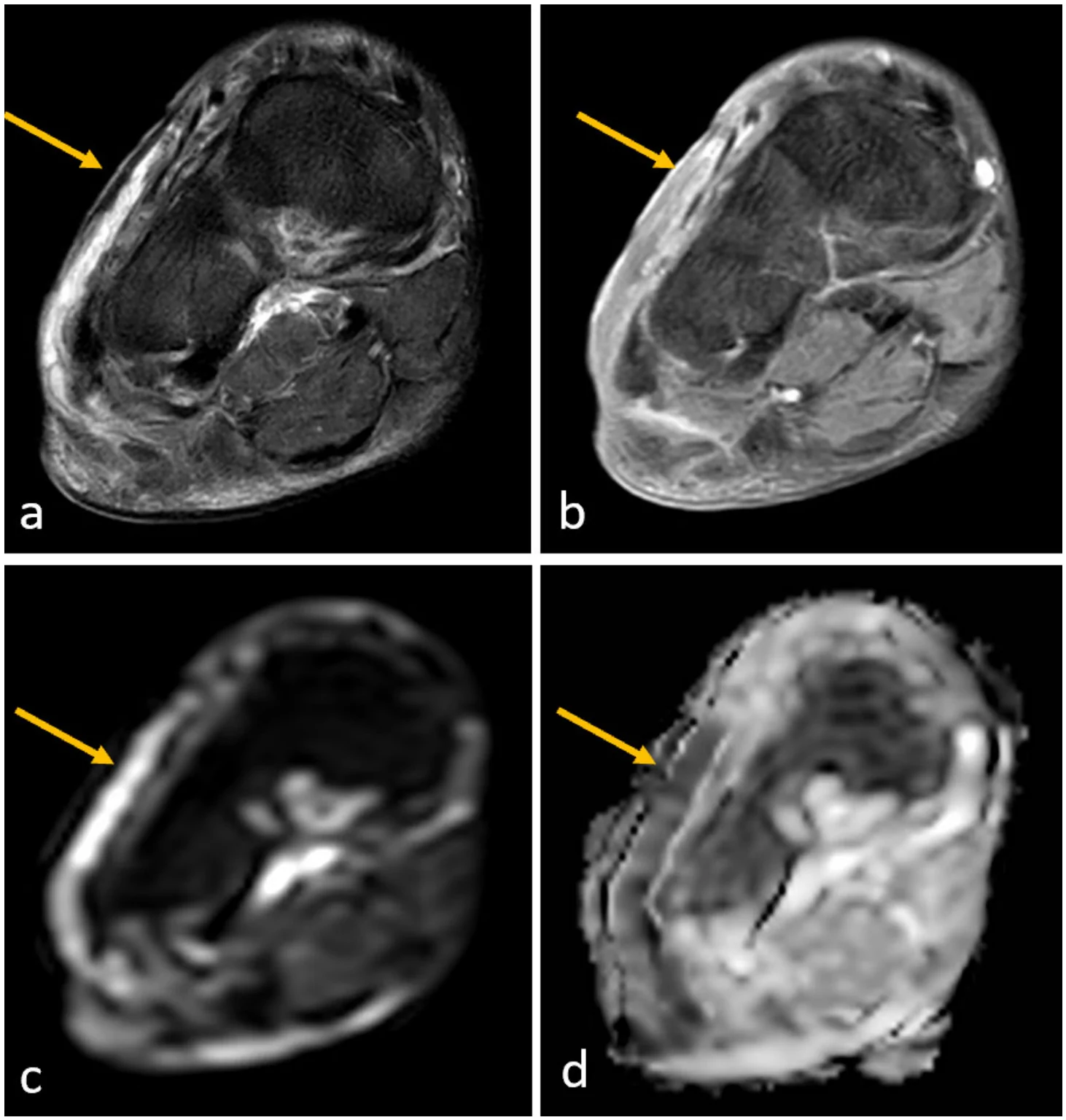
MRI showing improved assessment of an abscess on DWI (microbiology-proven). An ill-defined fat-suppressed T2-weighted hyperintense collection (**a**) is seen on the dorsal aspect of the foot and is not well appreciated on the T1-weighted post-contrast image (**b**). On DWI (**c**), the extent of the collection is better appreciated, with corresponding restricted diffusion on the ADC map (**d**) (orange arrows), suggesting that DWI provided better delineation of the collection than post-contrast imaging in this case. A single example cannot establish general superiority over contrast-enhanced imaging, and no comparative study data are cited here.

DWI has established itself as a clinically valuable tool for evaluating MSK infections, particularly when intravenous contrast administration is contraindicated [3,65]. DWI is particularly effective for detecting abscesses in devitalised tissue: purulent content restricts diffusion significantly, producing bright DWI signal with markedly low ADC values (Figure 15) (typically 0.6–1.1 × 10^−3^ mm^2^/s). Chun et al. demonstrated that DWI/ADC mapping at 3.0 T achieves diagnostic performance comparable to contrast-enhanced MRI for abscess detection, with mean ADC values of 0.88 × 10^−3^ mm^2^/s for abscesses, 2.2 × 10^−3^ mm^2^/s for phlegmons, and 3.1 × 10^−3^ mm^2^/s for bland oedema [23]. Harish et al. further confirmed that adding DWI to non-contrast MRI significantly improved diagnostic confidence in surgically proven abscesses [66].

Soft tissue oedema and cellulitis are frequently indistinguishable on conventional T1- and T2-weighted images but are readily differentiated by DWI. Bland oedema appears bright on both DWI and ADC maps (T2 shine-through; facilitated diffusion), whereas cellulitis appears bright on DWI with mild-to-moderately reduced ADC signal, reflecting the underlying inflammatory microenvironment [3,8]. A cut-off ADC of 1.2–2.0 × 10^−3^ mm^2^/s is commonly cited for differentiating cellulitis from simple oedema [65]. In postoperative patients, DWI helps differentiate seroma from an infective collection (abscess), with the former showing no restricted diffusion. The DWI and ADC characteristics across the spectrum of musculoskeletal infection are summarised in Table 7.

### 6.2. The ADC Ghost Sign in Osteomyelitis

**Figure 16:**
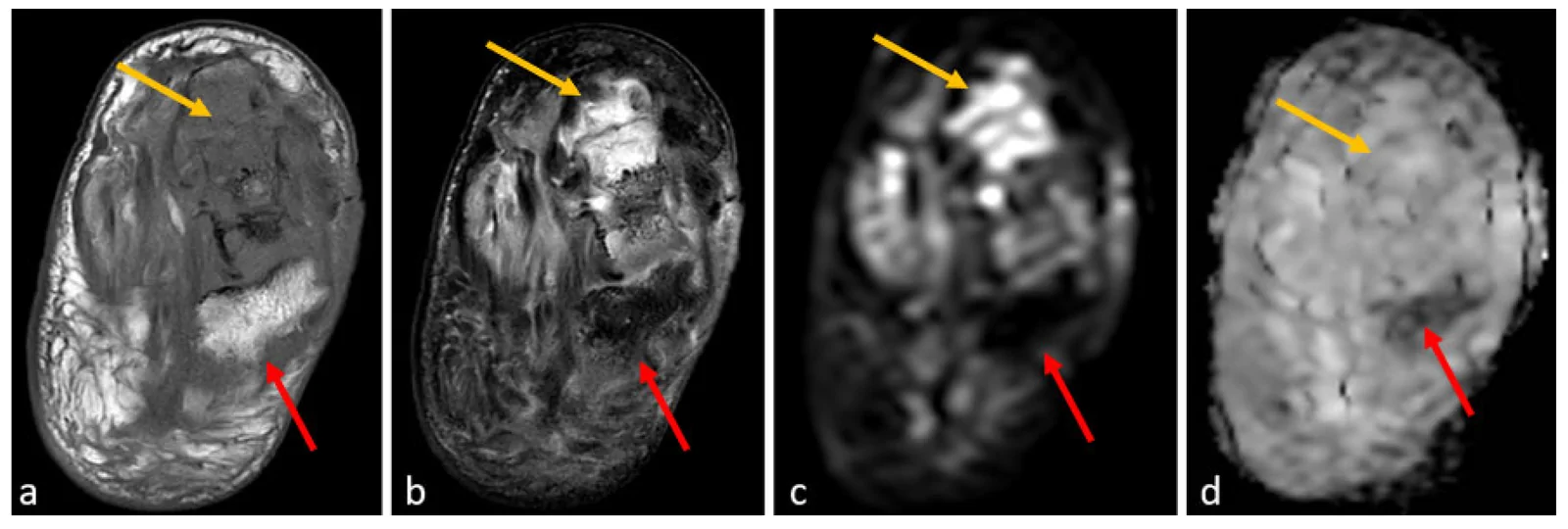
MRI of the foot demonstrating osteomyelitis of the talus (histopathology proven). Diffuse T1-weighted hypointense (**a**) and STIR hyperintense (**b**) marrow signal abnormalities are identified within the talus (orange arrows), with corresponding DWI hyperintensity (**c**). The ADC map (**d**) demonstrates complete loss of the normal marrow hypointensity, representing the ADC ghost sign. For comparison, normal adjacent bone (red arrows) demonstrates the expected T1-weighted hyperintense, STIR hypointense, and DWI- and ADC-hypointense marrow signal.

**Figure 17:**
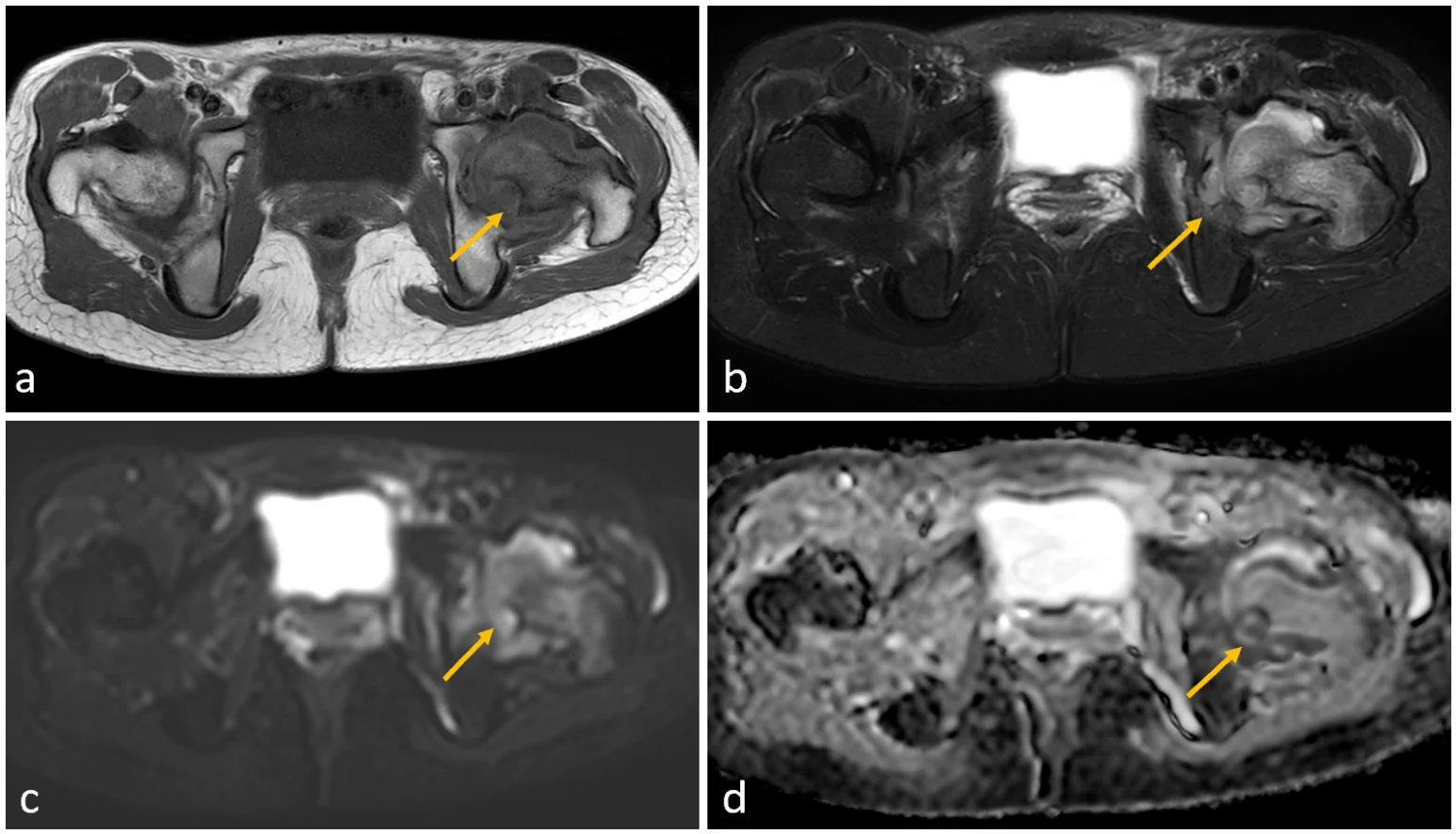
Axial MRI demonstrating septic arthritis with a moderate joint effusion on T1-weighted (**a**) and fat-suppressed T2-weighted (**b**) imaging. DWI (**c**) and the ADC map (**d**) demonstrate restricted diffusion of the inflamed synovial lining, with targeted synovial biopsy (microbiology proven) from the area of restricted diffusion confirming tubercular infection (Orange arrows). This case illustrates the expanding role of DWI beyond soft tissue abscess detection, providing functional localisation of active synovial inflammation in septic arthritis, guiding biopsy targeting, and ultimately contributing to a specific microbiological diagnosis.

The ADC ghost sign is a highly specific but insensitive marker for active osteomyelitis (OM) on DWI-generated ADC maps [25,67]. On fat-suppressed DWI, bone normally appears hypointense (dark) on ADC maps. In osteomyelitis, increased intramedullary water content from infection and reactive oedema replaces normal dark bone signal with diffuse high signal, while the associated periosteal and soft tissue reaction results in loss of normal bony cortical definition; the bone appears to “ghost” or dissolve into the surrounding inflamed tissue (Figure 16) [25]. He et al. described the ADC ghost sign as an early indicator of acute OM that may be visible before definitive changes on conventional T1 or T2 sequences, reporting it to be highly specific and more sensitive than the conventional T1-weighted penumbra or ghost signs [25]. It was reported that the sensitivities of the penumbra sign on T1-weighted imaging, the ghost sign on T1-weighted imaging, and the ghost sign on ADC maps for osteomyelitis were 3.7%, 9.8%, and 19.5%, respectively. In contrast, the corresponding specificities were markedly higher, at 98.9%, 97.8%, and 94.5%, respectively. Overall sensitivity of the ADC ghost sign remains modest at approximately 19–20%, though better than the other two MRI indicators of active osteomyelitis; however, its high specificity makes it a diagnostically valuable finding when present, particularly in the challenging differentiation of osteomyelitis from Charcot neuroarthropathy in the diabetic foot [25,67]. It should be emphasised that a sensitivity of this order means the sign is absent in roughly four of five cases of proven osteomyelitis. The ghost sign is therefore a rule-in finding only; its absence carries almost no negative predictive value, and it cannot be described as an established means of early diagnosis. The supporting evidence derives from a single retrospective series and has not been externally validated. DWI also plays a role in distinguishing osteomyelitis from septic arthritis (Figure 17) and in evaluating complex musculoskeletal infections in anatomically challenging regions such as the foot and ankle [26].

## 7. Whole-Body DWI: Neurocutaneous Syndromes and Paediatric Applications

**Evidence tier.** Tier 3 for neurocutaneous and paediatric cancer predisposition surveillance, where cohorts are small and no outcome data are available (Table 5).

WB-MRI is the modality of choice for evaluating peripheral nerve sheath tumour (PNST) burden in neurocutaneous syndromes, including NF1, NF2, and schwannomatosis [27,59]. Adding DWI with ADC mapping to WB-MRI substantially improves the characterisation of individual lesions: it aids in detecting malignant transformation within PNSTs, differentiates perineural cysts from true nerve tumours, and enables ADC-based grading of lesion cellularity across multiple body regions in a single examination [27]. In NF1, DWI is especially valuable given the approximately 10% lifetime risk of malignant transformation of plexiform neurofibromas to MPNSTs, where a minimum ADC ≤ 1.0 × 10^−3^ mm^2^/s serves as a key indicator of malignancy [27,29]. For paediatric cancer predisposition syndromes such as Li–Fraumeni syndrome, WB-MRI with DWI/ADC offers a radiation-free surveillance strategy that may serve as a viable alternative to radiography, CT, and 18F-FDG PET/CT [59]. Although not yet fully validated for all these indications in prospective cohorts, the absence of ionising radiation represents a compelling advantage in children and young adults requiring long-term surveillance [59].

## 8. DWI in Inflammatory Myopathies

**Evidence tier.** Tier 3. Supporting studies are small and heterogeneous, progressive fatty infiltration confounds ADC in chronic disease, and no threshold has been externally validated. DWI should be regarded as investigational in this setting (Table 5).

Conventional MRI plays a central role in diagnosing and monitoring idiopathic inflammatory myopathies (IIMs) such as polymyositis and dermatomyositis; however, there is considerable overlap in appearances across different myopathy subtypes: STIR sequences show non-specific intramuscular oedema-like signal, while T1-weighted sequences reflect progressive fatty replacement and atrophy [30,31]. This limits diagnostic specificity and hampers objective disease activity monitoring.

DWI with ADC mapping is being actively investigated as a functional complement. Qi et al. demonstrated the feasibility of DWI in IIMs, finding elevated ADC and true diffusion coefficient (D) values in inflamed muscles compared to unaffected contralateral muscles, while fatty-replaced muscles demonstrated the lowest ADC values [30]. Faruch et al. confirmed a positive correlation between ADC values and the grade of muscle oedema in IIM patients, with DWI detecting muscle oedema even before changes became apparent on STIR sequences [31]. Saran et al. additionally demonstrated significantly higher average ADC, FA, and eigenvalues in IIM patients compared to healthy controls on DTI analysis of the vastus lateralis muscle [32]. The ADC profile across muscle states in inflammatory myopathy is summarised in Table 8.

A key technical consideration in chronic myopathy is that progressive intra- and intermuscular fat accumulation produces spuriously low ADC values, a pitfall that must be recognised in DWI interpretation [30]. The potential role of DWI as a quantitative biomarker for disease severity and treatment response in IIMs is promising but requires validation in prospective cohorts, and no studies have yet established it as a validated treatment monitoring tool in this setting [32].

## 9. DWI in the Evaluation of Nerves, Neuropathies and Plexopathies

**Evidence tier.** Tier 3. Nerve DWI is limited by low signal-to-noise ratios in small-calibre structures and by susceptibility artefacts, and current evidence does not establish that ADC reliably differentiates between neuropathies. The applications described here are investigational (Table 5).

DWI, including single-b-value acquisitions and whole-body DWI with background fat and vascular suppression, has significantly improved the conspicuity of peripheral nerves, particularly in complex anatomical regions such as the brachial plexus [33,34]. Suppression of vascular signal at higher b-values enhances nerve visualisation and facilitates more consistent interpretation, contributing to improved interobserver agreement, especially in the assessment of oncologic plexopathies.

Despite these advantages, DWI of peripheral nerves remains technically challenging. Small nerve calibre contributes to an inherently low signal-to-noise ratio (SNR), which can limit image quality. Although high-field MRI systems improve the SNR, they are also associated with increased susceptibility artefacts, particularly at air–tissue interfaces, which may degrade image fidelity [34]. Quantitatively, normal peripheral nerves demonstrate relatively consistent apparent ADC values. For example, the median nerve has been reported to exhibit an ADC of approximately 1.01 ± 0.13 × 10^−3^ mm^2^/s under normal conditions [33]. This is lower than the indicative range given for peripheral nerves in Table 3, which is drawn from a different source; as discussed in Section 2.3, the nerve ADC is particularly sensitive to partial volume effects given the small calibre of the structures involved, and site-specific and protocol-specific reference values are required before deviations can be interpreted as pathological.

In inflammatory neuropathies such as chronic inflammatory demyelinating polyneuropathy (CIDP), increased ADC values have been observed compared to healthy controls (1.27 ± 0.43 vs. 0.92 ± 0.11 × 10^−3^ mm^2^/s). These changes are thought to reflect a combination of T2 shine-through effects and structural alterations such as nerve hypertrophy. However, current evidence remains limited regarding the ability of DWI and ADC metrics to reliably differentiate CIDP from other neuropathic conditions, including Charcot–Marie–Tooth disease, monoclonal gammopathy-associated neuropathy, and multifocal motor neuropathy [34].

DWI also shows promise in distinguishing inflammatory plexopathies from infiltrative or metastatic disease, where differences in diffusion characteristics and structural involvement may aid diagnosis. In addition, the technique enhances visualisation of nerve architecture and pathology, including thickening and hyperintensity of affected nerves, as seen in demyelinating plexopathies. Advanced diffusion techniques, such as DTI, extend the capabilities of conventional DWI by enabling evaluation of anisotropy and nerve fibre organisation [5,34]. These methods may provide further insights into microstructural integrity and disease-related alterations in peripheral nerves. Secondary findings associated with neuropathic processes, such as denervation oedema and muscle atrophy, can also be detected on DWI. For instance, in cases of plexopathy, affected muscles may demonstrate signal changes consistent with denervation, including oedema and subsequent atrophy.

Overall, DWI and its advanced derivatives represent valuable adjuncts in the evaluation of peripheral nerve and plexus disorders, although further studies are needed to refine their diagnostic specificity and clinical utility.

## 10. DWI as an Opportunistic Tool: Detection and Staging of Deep Vein Thrombosis

**Evidence tier.** Tier 3. Incidental detection of thrombus during a musculoskeletal examination is clinically useful and worth reporting. ADC-based estimation of thrombus age rests on a single small cohort, has not been externally validated, and does not determine management (Table 5).

**Figure 18:**
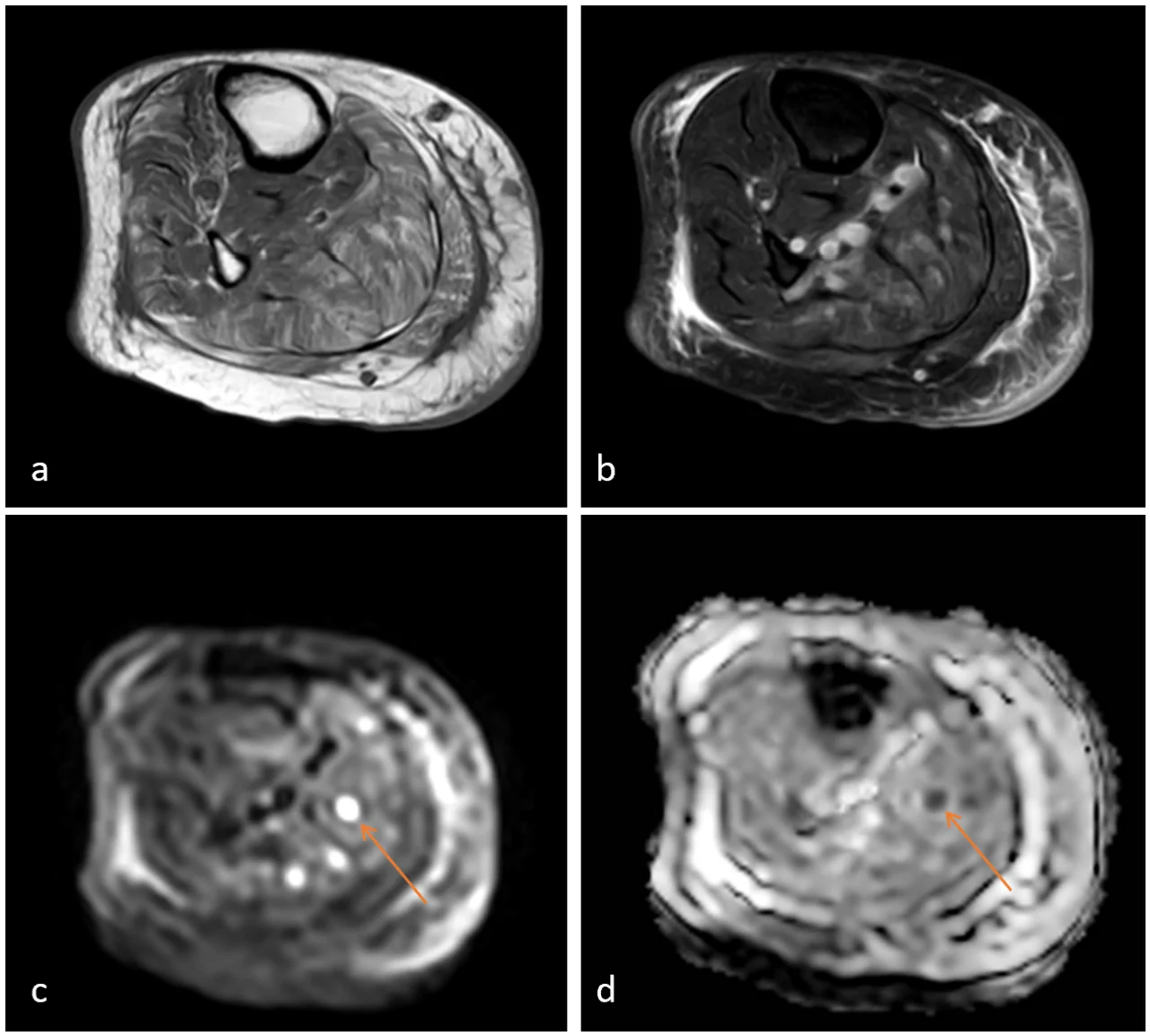
Axial MRI of a patient with cellulitis demonstrating an incidentally detected acute soleal vein thrombosis (image-based diagnosis only). The acute thrombus within the soleal vein is inconspicuous on T1- and T2-weighted sequences (**a**,**b**) but is clearly identified as a focus of high signal on DWI with corresponding restricted diffusion on the ADC map (**c**,**d**) (orange arrows), highlighting the additive value of DWI in the detection of acute deep vein thrombosis.

**Figure 19:**
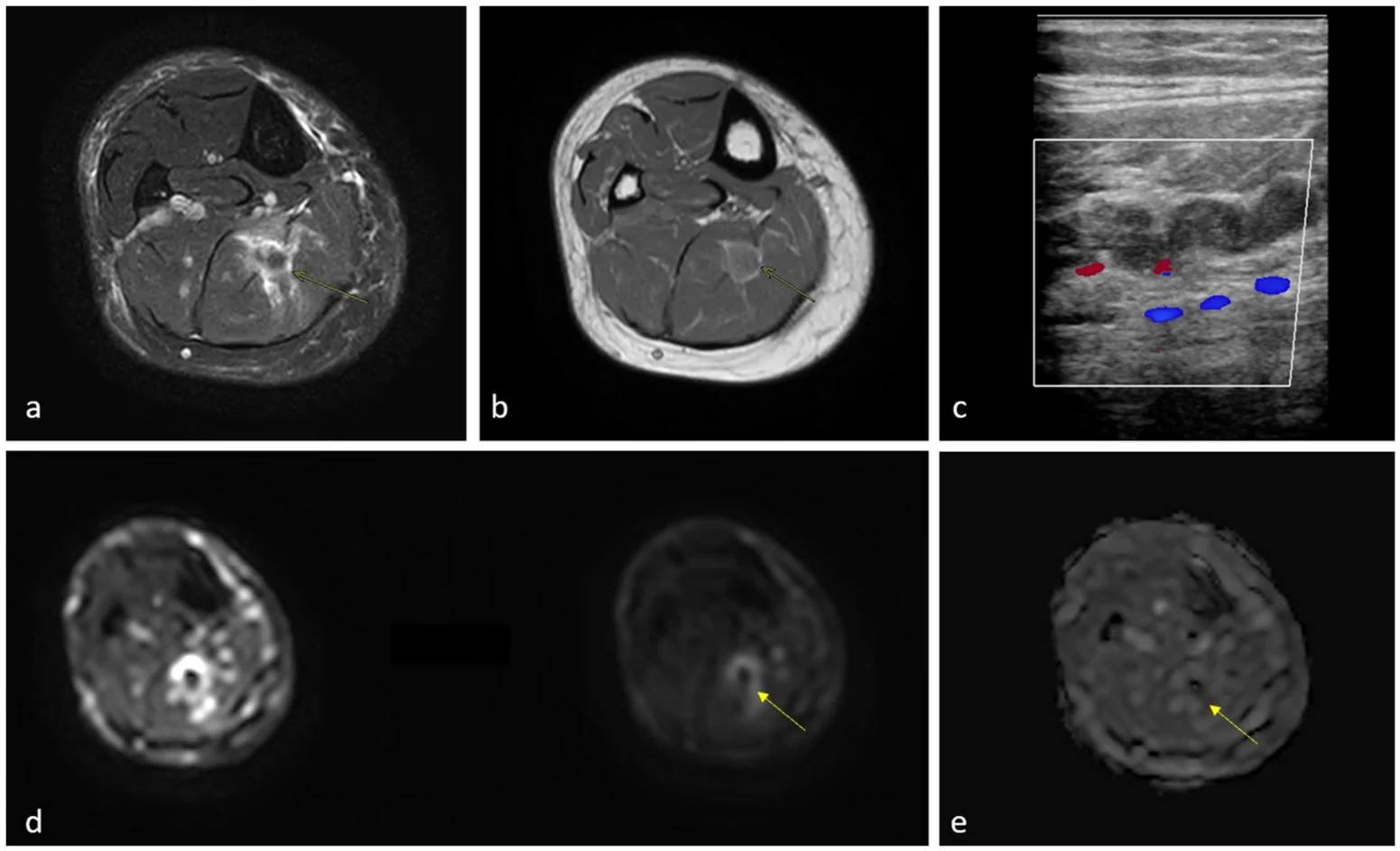
Axial MRI demonstrating soleal vein thrombosis in a patient with a sports injury suspected of a muscle tear. T2-weighted imaging shows persistent expansion of the soleal vein with hypointense intraluminal signal (**a**) and hyperintense signal on the T1-weighted image with luminal expansion (**b**), with correspondingly low signal on DWI and the ADC map (**d**,**e**) (yellow arrows), in keeping with non-acute (subacute) thrombosis (image-based diagnosis only). Targeted colour Doppler ultrasound (white box) showed no colour flow confirming the diagnosis (**c**). This demonstrates the diagnostic value of DWI in the detection of deep vein thrombosis.

An emerging and clinically significant application of DWI in MSK imaging is its opportunistic role in detecting and characterising deep vein thrombosis (DVT). Colour Doppler ultrasound remains the primary investigation for suspected DVT, with sensitivity and specificity exceeding 95% for proximal thrombus; however, it is operator-dependent, poorly evaluates iliac veins and deep calf veins in the setting of oedema, and is unable to characterise thrombus age or internal structure [46]. DWI acquired as part of routine lower limb MSK examination can serve as an opportunistic screening tool for DVT, with the additional advantage of providing information on thrombus age, a distinction that is clinically relevant in selected patients (Figure 18 and Figure 19). It should be stated at the outset that this is an opportunistic adjunct and nothing more. DWI performed for a musculoskeletal indication may draw attention to a thrombus that would otherwise have gone unremarked, and that alone is worth reporting. It does not replace compression ultrasound, clinical probability scoring, D-dimer testing or dedicated venous imaging; it has not been evaluated as a screening test in an unselected population, and a negative DWI examination does not exclude deep vein thrombosis.

### Pathophysiology Underpinning DWI Differentiation of Acute and Non-Acute DVT

Thrombi undergo time-dependent organisation involving endothelialisation, macrophage infiltration, fibroblast activity, and progressive fibrosis, all of which influence imaging characteristics [35]. For consistency, thrombi are described here as acute (approximately <14 days) or non-acute (approximately >14 days), the latter encompassing both subacute and chronic organised thrombus; these intervals are approximations rather than biological boundaries. Acute thrombus generally has higher water content and yields relatively high DWI signal (Figure 18). The role of methaemoglobin in this appearance requires qualification: blood product evolution is a continuum, and deoxyhaemoglobin, intracellular and extracellular methaemoglobin and haemosiderin coexist in varying proportions that change over hours to weeks and differ between and within thrombi. Methaemoglobin is therefore not a uniform property of all thrombi under 14 days old, and its paramagnetic T2-shortening effect competes with the T2-prolonging effect of high water content, so that the signal at any given time point reflects the balance of the two rather than a single dominant mechanism. Non-acute thrombus shows reduced water content and progressive fibrous organisation, narrowing the space available for water diffusion and producing lower DWI signal and lower ADC values [35] (Figure 19).

Published data demonstrate significantly different ADC values between acute and non-acute thrombi: 0.56 ± 0.17 × 10^−3^ mm^2^/s for acute versus 0.22 ± 0.12 × 10^−3^ mm^2^/s for non-acute DVT, with ADC achieving 93% sensitivity and 90% specificity for discrimination between the two [35]. These figures come from a small single-centre study and should be read with corresponding caution. The manuscript reporting them does not provide external validation, and the published account gives limited detail on patient selection, on the reference standard used to establish thrombus age, on whether observers were blinded, and on confidence intervals around the reported sensitivity and specificity. The apparent separation between groups is also narrower in practice than the point estimates suggest since the reported standard deviations overlap. No prospective or multicentre validation of ADC-based thrombus ageing has been published, and the technique should be regarded as investigational. The DWI and ADC characteristics distinguishing acute from non-acute thrombus are summarised in Table 9.

Any inference from thrombus age to treatment must be made with equal caution. The great majority of deep vein thrombi are managed with anticoagulation alone. Catheter-directed thrombolysis and mechanical thrombectomy are reserved for selected situations, principally extensive iliofemoral thrombosis with severe symptoms, phlegmasia cerulea dolens, or limb-threatening presentations in patients with acceptable bleeding risk, and their role remains contested. Duration of anticoagulation is determined by whether the event was provoked or unprovoked, by thrombus location and extent, and by recurrence risk, bleeding risk, malignancy and patient preference. None of these decisions can be made from an ADC value. The appropriate contribution of DWI is to raise the possibility of thrombus and to offer a provisional impression of its age, which is then confirmed and acted upon through established vascular imaging and clinical pathways.

## 11. Technical Pitfalls in DWI

DWI imaging pitfalls fall into two broad categories: technical and interpretation-related. A paradigm shift in MSK DWI practice is reframing many traditional “pitfalls” not simply as limitations but as biologically informative phenomena that can be strategically leveraged for diagnostic gain when properly understood [3,8].

Susceptibility artefacts arise from magnetic susceptibility differences at tissue interfaces—bone, soft tissue, air, and metallic implants—causing geometric distortion and signal loss [3,40]. Mitigation strategies include multi-shot EPI, higher bandwidth, parallel imaging, and non-EPI DWI techniques such as PROPELLER, BLADE, or RESOLVE [3,40]. The use of an external permanent magnet to correct field distortions near metallic hardware has been described as a recent advance [8]. Fat suppression failures near bone marrow and implants can obscure pathology; STIR-based fat suppression provides more uniform performance in regions of field inhomogeneity, and Dixon-based fat/water separation is a further option in challenging anatomical locations [3].

Motion artefacts from voluntary patient movement or physiological motion (cardiac pulsation, respiratory movement) degrade image quality; single-shot EPI, navigator-based motion correction, and ECG/respiratory gating reduce this problem [3]. Partial volume effects are a concern for small lesions near bone or muscle boundaries, addressable by thin slices (≤4 mm) and correlation with anatomical sequences [3]. B-value standardisation across institutions remains an unresolved challenge impeding multicentre ADC comparability; phantom-based ADC calibration and standardised protocols (b = 0, 50, 400, 800 s/mm^2^) are recommended [3,8].

## 12. Composition-Related Interpretive Pitfalls

The scenarios collected in this section are conventionally described as interpretation pitfalls. They are presented here instead as the clinical expression of the framework set out in Section 3: each represents a lesion in which composition, rather than cellularity, dominates the diffusion signal. They are therefore organised by substrate rather than by the name of the artefact, since it is the substrate—not the phenomenon—that the radiologist must infer at the workstation. Each subsection states the mechanism, illustrates it with an institutional case, and closes with the practical rule that follows.

### 12.1. Free Water and Prolonged T2: Myxoid Matrix and Cystic Lesions

T2 Shine-Through: The T2 shine-through effect causes lesions with high intrinsic T2 signal (ganglion cysts, oedema, fluid-filled, myxoid lesions) to appear bright on DWI, mimicking restricted diffusion. Identification and correction require reviewing ADC maps; true restriction shows low ADC, T2 shine-through shows correspondingly high ADC. Clinically, this phenomenon differentiates synovial osteochondromatosis (T2 shine-through, high ADC) from PVNS (T2 black-through, low ADC) [3,8].

**Figure 20:**
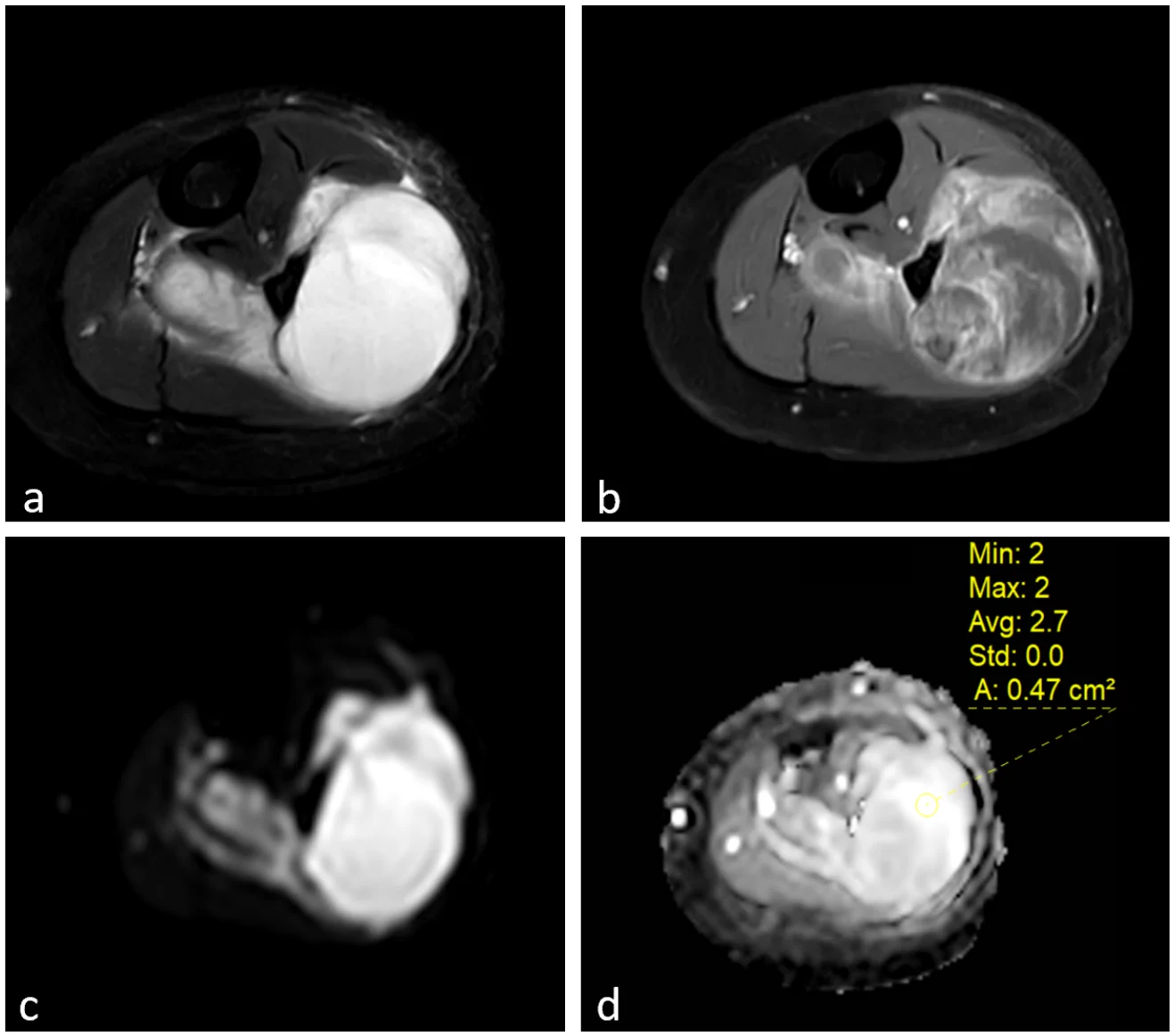
Axial MRI of a biopsy-proven myxofibrosarcoma (histopathology-proven) of the leg, demonstrating T2-weighted hyperintensity (**a**), heterogeneous post-contrast enhancement on T1-weighted imaging (**b**), and high signal on DWI (**c**). Despite its malignant nature, the ADC map (**d**) shows no convincing restricted diffusion, which is a recognised pitfall attributable to the abundant myxoid matrix, which prolongs T2 relaxation and produces T2 shine-through, thereby masking true restriction and falsely suggesting a benign lesion.

**Figure 21:**
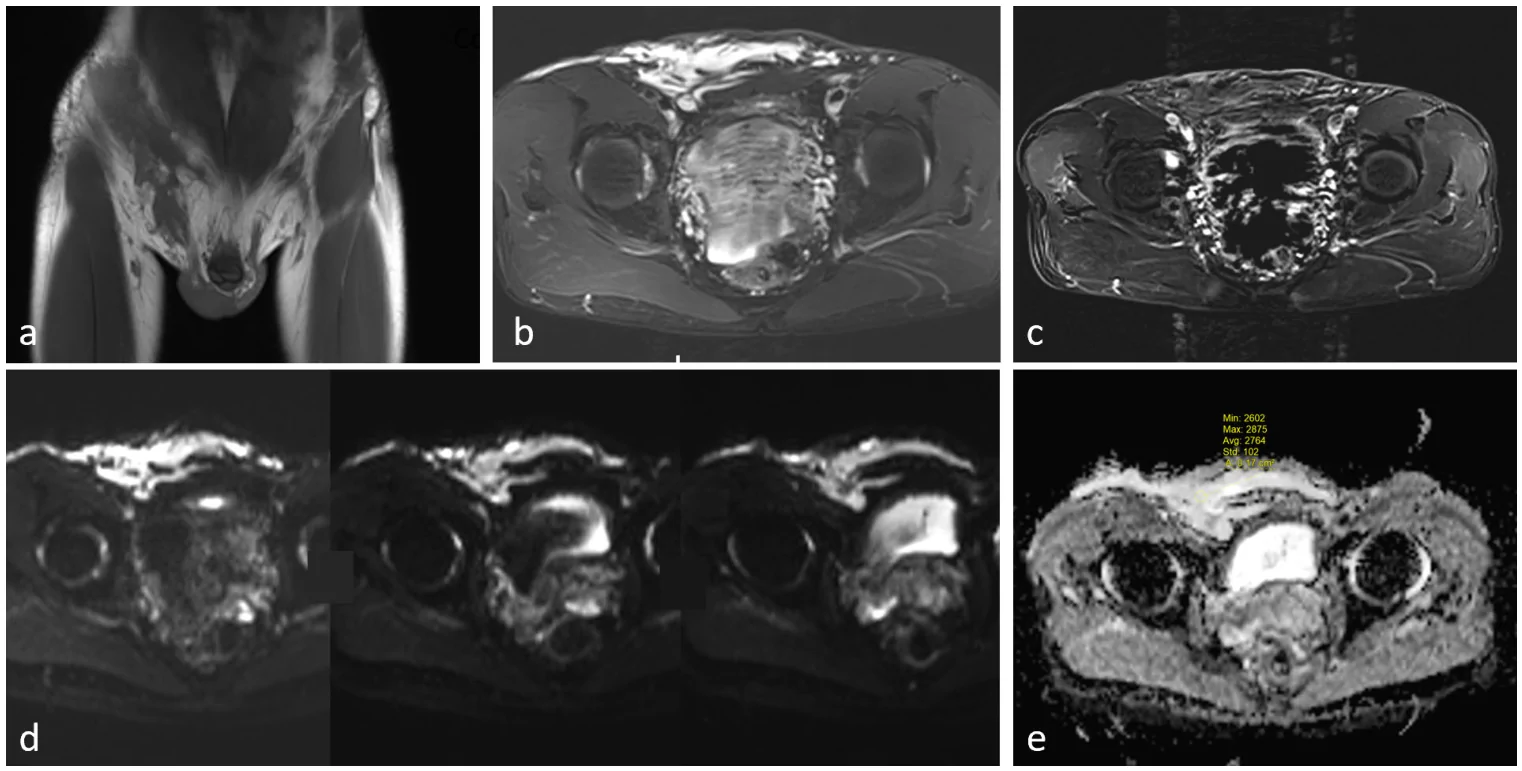
Axial MRI of a benign aggressive angiomyxoma (histopathology-proven) demonstrating an ill-defined, insinuating T1-weighted hypointense (**a**) and fat-suppressed T2-weighted hyperintense (**b**) mass in the right inguinal region, with faint post-contrast enhancement (**c**). On DWI (**d**), the lesion shows hyperintensity at low b-values with progressive signal loss at higher b-values, and the ADC map (**e**) demonstrates no focus of restricted diffusion—consistent with T2 shine-through rather than true restriction, in keeping with the benign nature of the lesion.

Malignant myxoid tumours paradoxically demonstrate high apparent diffusion coefficient (ADC) values on diffusion-weighted imaging (DWI), despite the general expectation that malignant lesions should exhibit diffusion restriction and correspondingly low ADC values. For example, myxofibrosarcoma can show ADC values within a range similar to those observed in benign lesions such as angiomyxoma, as shown (Figure 20 and Figure 21).

This phenomenon is primarily attributable to the unique histological composition of myxoid tumours. The myxoid extracellular matrix is rich in free water content, with relatively low collagen density and a loose stromal architecture. These features facilitate increased water proton mobility, resulting in elevated ADC values, often exceeding 2.5 × 10^−3^ mm^2^/s.

In addition, myxoid tissues typically exhibit prolonged T2 relaxation times. This can lead to the T2 shine-through effect, whereby high signal intensity on DWI is not due to true diffusion restriction but rather reflects intrinsic T2 hyperintensity, potentially causing overestimation of ADC values.

Given these factors, it is important to recognise the limitations of DWI and ADC interpretation in myxoid tumours. Accurate assessment should rely on a multiparametric MRI approach, incorporating correlation with T1- and T2-weighted imaging characteristics, contrast enhancement patterns, lesion morphology, and relevant clinical information to avoid diagnostic pitfalls.

### 12.2. Haemosiderin and Fibrocollagenous Matrix: The T2 Black-Through Signature

**Figure 22:**
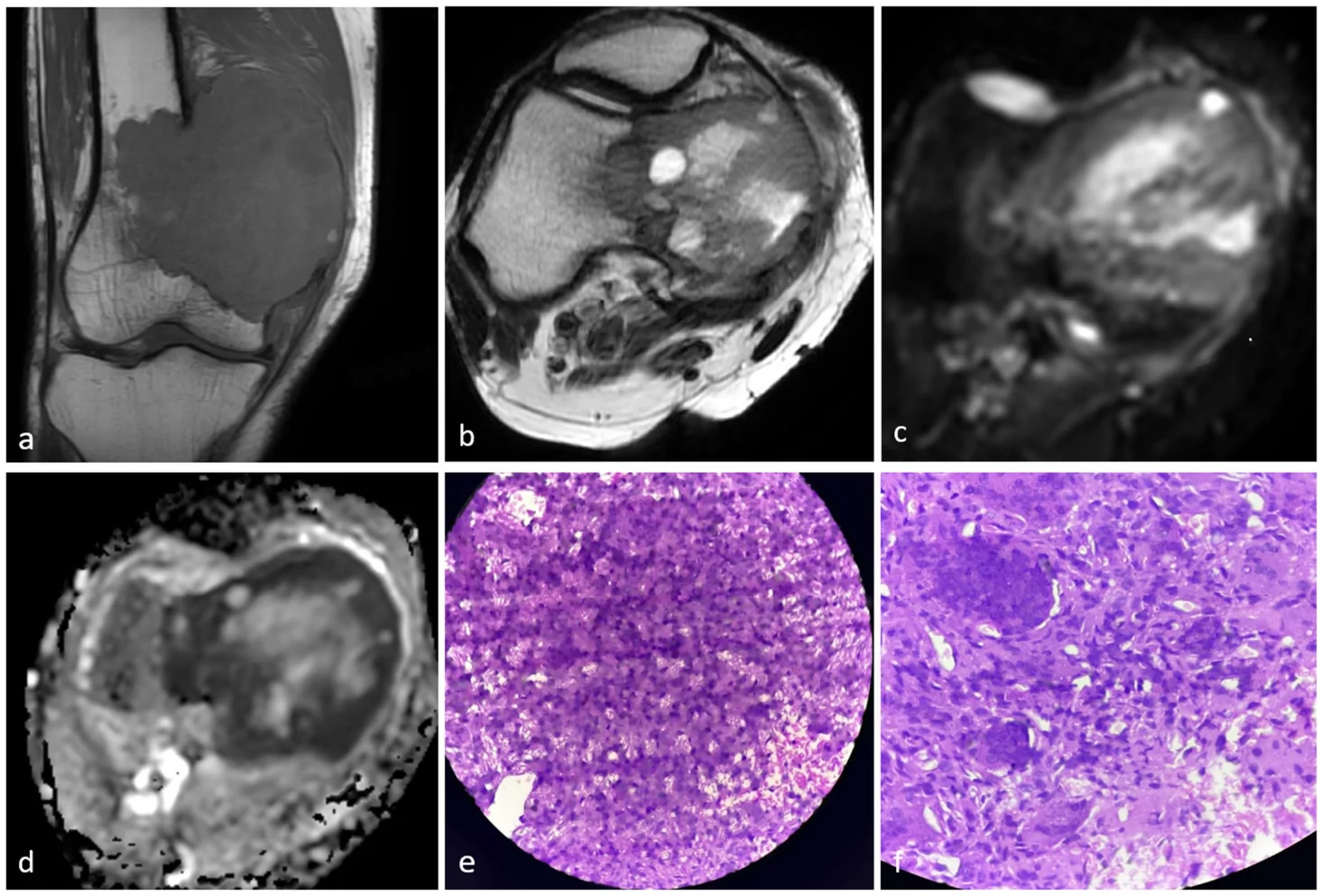
MRI of a histologically proven giant cell tumour of the distal femur. The lesion is hypointense on the coronal T1-weighted image (**a**). On T2-weighted imaging (**b**), it shows a peripheral hypointense rim with central heterogeneously hyperintense areas. On diffusion-weighted imaging (**c**), the peripheral component appears hypointense and shows spuriously low signal on the corresponding ADC map (**d**), mimicking restricted diffusion (T2 blackout effect)—a potential interpretive pitfall. Histology slides (**e**,**f**) show osteoclastic giant cells surrounded by mononuclear cells. These cells are spindled with elongated nuclei and moderate cytoplasm. Image courtesy Dr. Suvinay Saxena.

T2 Black-Out/Black-Through Effect: Tissues with intrinsically low T2 signal—such as hemosiderin deposits (PVNS, giant cell tumour), fibrous tissues (mature desmoid), calcifications and osteoid matrix—appear dark on both DWI and ADC maps. Reframed diagnostically, the T2 black-through effect reliably identifies hemosiderin-laden pathologies and fibrous tumours (Figure 22), adding diagnostic confidence rather than causing confusion [3,8,12].

### 12.3. Blood Degradation Products: Haematoma as Tumour and Abscess Mimic

**Figure 23:**
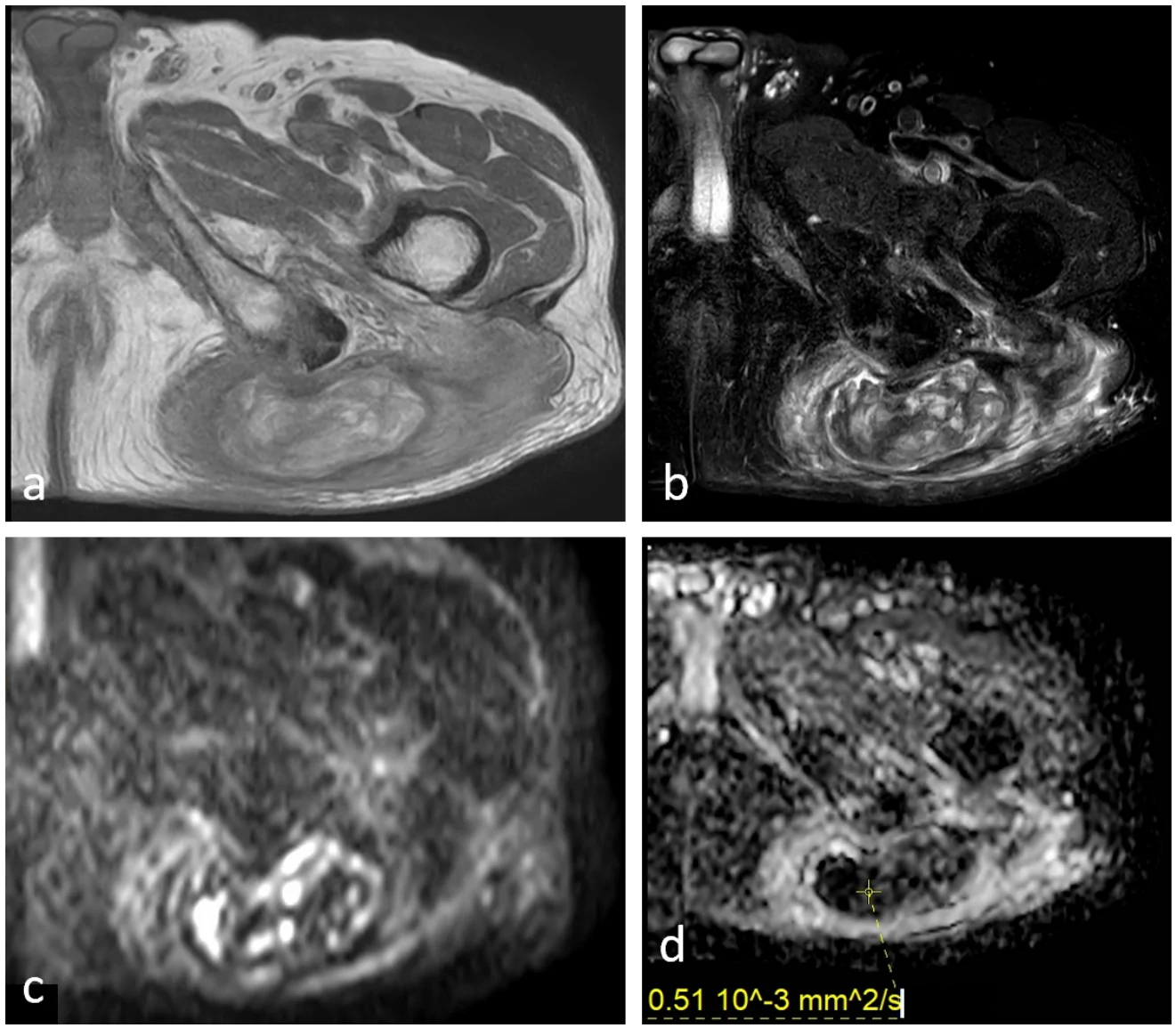
Axial MRI of a left gluteal haematoma (image-based diagnosis only) demonstrating a T1-weighted hyperintense (**a**) and T2-weighted hypointense (**b**) collection within the gluteus maximus muscle. The collection is hypointense on both DWI (**c**) and the ADC map (**d**). This combination is not the conventional pattern of restricted diffusion, which requires high signal on high-b-value DWI together with low ADC. Here, the markedly shortened T2 of the blood products suppresses the signal on the diffusion-weighted images themselves, and the low ADC is a consequence of fitting a decay curve to an already noise-limited signal rather than evidence of impeded water motion. The practical rule is that low ADC should be accepted as genuine restriction only when the corresponding high-b-value DWI is bright; where both are dark, the underlying substrate is haemorrhagic, fibrous, crystalline or mineralised, and the ADC value should not be interpreted quantitatively.

A haematoma represents a well-recognised tumour mimic on diffusion-weighted imaging (DWI) and apparent diffusion coefficient (ADC) mapping and remains an important diagnostic pitfall in both primary and postoperative imaging settings (Figure 23). Depending on its stage of evolution, a haematoma may closely resemble a soft tissue neoplasm or abscess, particularly when it demonstrates restricted diffusion. This overlap arises from the complex biochemical and structural changes that occur during blood degradation, including intracellular methaemoglobin formation, increased viscosity, and reduced extracellular water mobility, all of which can contribute to low ADC values.

Restricted diffusion within hematomas can be particularly misleading, as it may simulate the appearance of a pyogenic abscess, where central diffusion restriction is typically attributed to purulent material and high cellularity. Similarly, haemorrhagic components within tumours may artificially reduce ADC values, leading to overestimation of tumour cellularity or erroneous classification as infection. These pitfalls are especially relevant when ADC measurements are obtained without careful attention to lesion heterogeneity, as sampling haemorrhagic regions may not reflect the true diffusion characteristics of the underlying pathology.

The variability in ADC values in hematomas has been well documented and largely reflects the age of the haemorrhage. Acute and early subacute hematomas tend to demonstrate lower ADC values, often overlapping with those of malignant soft tissue tumours. For example, Subhawong et al. reported very low ADC values in hematomas, with a minimum of 0.56 × 10^−3^ mm^2^/s and a mean of 0.82 × 10^−3^ mm^2^/s, values that significantly overlap with those observed in soft tissue sarcomas [10]. In contrast, more chronic hematomas may exhibit higher ADC values due to liquefaction and breakdown of clot structure. Oka et al., in their study of chronic expanding hematomas, reported a mean ADC of 1.55 ± 0.12 × 10^−3^ mm^2^/s, significantly higher than that of malignant soft tissue tumours (0.92 ± 0.14 × 10^−3^ mm^2^/s, *p* < 0.01) [36]. Similarly, del Grande et al. found even higher ADC values in hematomas, with a minimum of 1.78 ± 0.71 × 10^−3^ mm^2^/s and a mean of 2.34 ± 0.72 × 10^−3^ mm^2^/s, exceeding those of recurrent neoplasms (minimum 0.97 ± 0.13 × 10^−3^ mm^2^/s; mean 1.08 ± 0.19 × 10^−3^ mm^2^/s) [11].

These findings underscore the significant overlap in ADC values between hematomas and soft tissue neoplasms, as well as the dependence of diffusion characteristics on the temporal evolution of haemorrhage. The diagnostic challenge is further compounded in the postoperative setting, where the differential diagnosis often includes recurrent tumour, postoperative haematoma, and infection. In such contexts, mixed signal intensities from blood products, inflammatory changes, and granulation tissue can confound interpretation and lead to misdiagnosis if DWI is assessed in isolation.

Accordingly, diffusion imaging must always be interpreted in conjunction with conventional anatomic MRI sequences. Correlation with T1-weighted, T2-weighted, and gradient sequences is essential to identify blood products and characterise their stage of evolution. Additional features such as lesion morphology, internal architecture, presence of a capsule, surrounding oedema, and contrast enhancement patterns provide indispensable context. Only through a multiparametric approach can radiologists reliably distinguish haematomas from soft tissue neoplasms and abscesses, thereby avoiding the significant diagnostic pitfalls associated with DWI and ADC interpretation alone.

### 12.4. Crystalline and Mineralised Material: The Gouty Tophus

**Figure 24:**
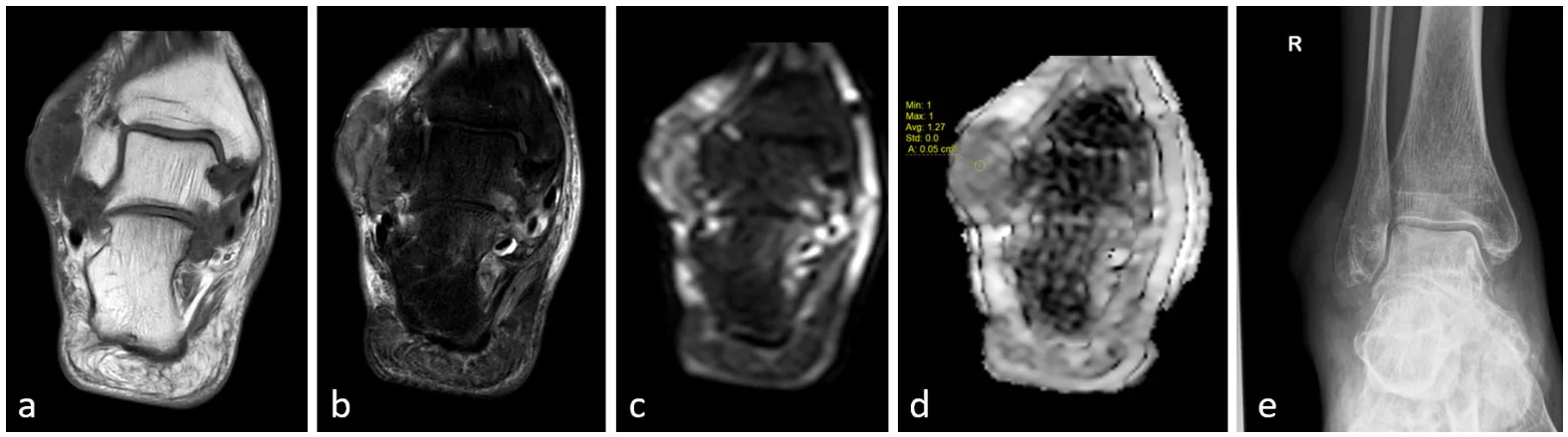
Coronal MRI and radiograph of the ankle demonstrating a gouty tophus. The lesion presents as a lobulated mass on the lateral aspect of the ankle, appearing as predominantly T1-weighted hypointense (**a**) and fat-suppressed T2-weighted hypointense (**b**). Importantly, whilst the ADC map (**d**) demonstrates apparently low signal, suggesting restricted diffusion, DWI (**c**) shows no corresponding hyperintensity—a recognised pitfall in which spuriously low ADC signal in the absence of DWI hyperintensity may be erroneously interpreted as true restricted diffusion. The frontal radiograph (**e**) demonstrates a lobulated soft tissue swelling with internal foci of calcification, characteristic of gouty tophi.

Gouty tophi, characterised by deposition of monosodium urate crystals, represent an important diagnostic pitfall in DWI (Figure 24). The lesion in our case exhibits low signal intensity on T2-weighted imaging, as well as low signal on both DWI and ADC maps. This signal profile likely reflects the presence of dense crystalline material, calcification, and fibrotic components, all of which contribute to reduced proton mobility and intrinsic low signal rather than true diffusion restriction. The corresponding calcifications seen on radiography further support this composition and explain the signal suppression across sequences. Such findings may lead to diagnostic confusion, as the combination of T2 hypointensity and DWI hypointensity can mimic entities characterised by mineralisation or haemosiderin deposition, rather than the more typical infective or hypercellular lesions associated with diffusion restriction. This case underscores the importance of recognising that gouty tophi may demonstrate variable DWI characteristics depending on their internal composition and highlights the need for correlation with radiographic findings to avoid misinterpretation.

### 12.5. Keratinaceous and Viscous Contents: Benign Lesions That Restrict

**Figure 25:**
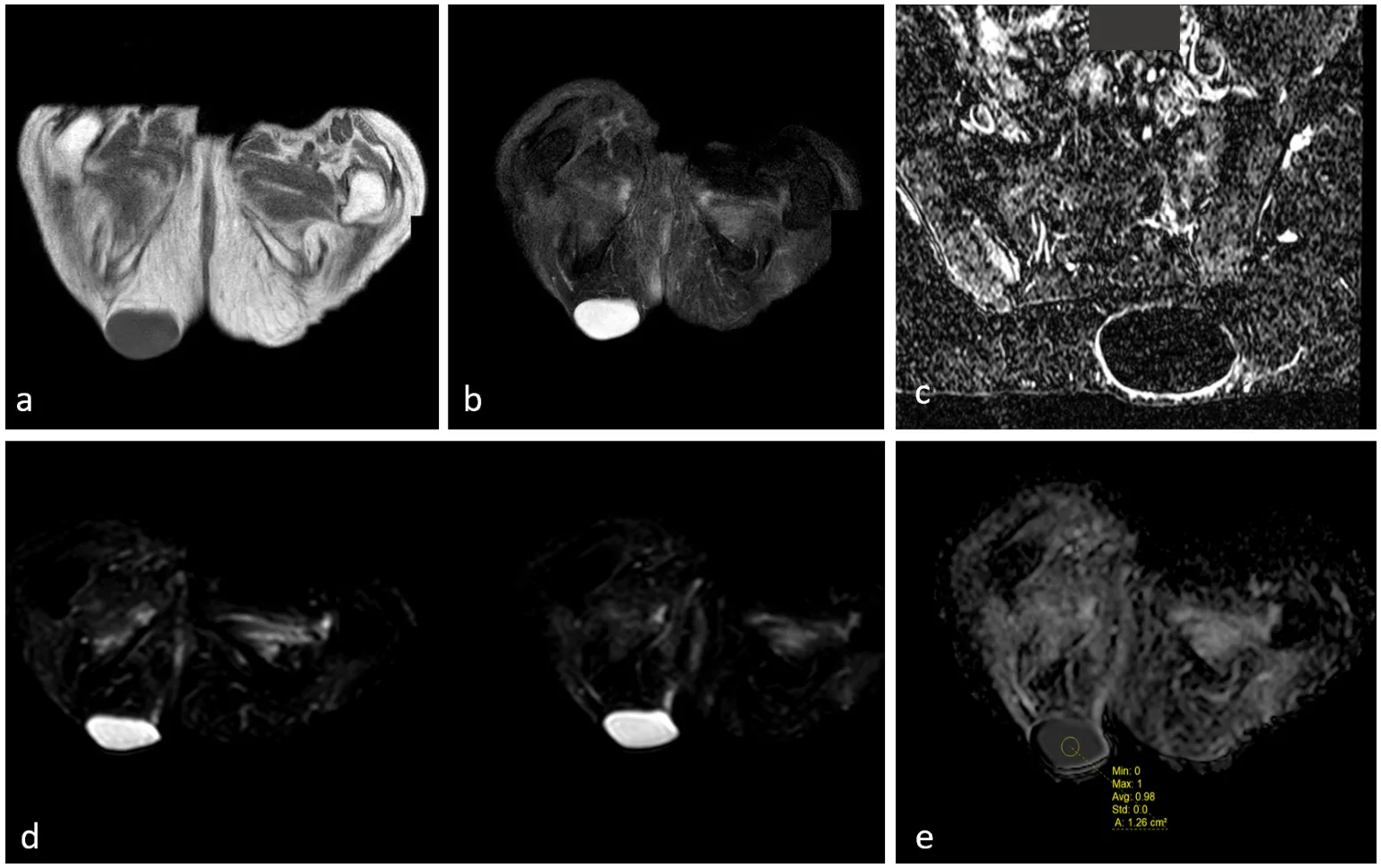
Axial MRI of an epidermoid cyst demonstrating T1-weighted hypointense (**a**) and T2-weighted hyperintense (**b**) signal with thin peripheral enhancement on subtracted post-contrast T1-weighted imaging (**c**). Despite its benign nature, the lesion shows high signal on DWI (**d**), with corresponding restricted diffusion on the ADC map (**e**), representing a potential diagnostic pitfall. This appearance is attributed to the viscous internal contents of the cyst, including keratin, cholesterol, and proteinaceous debris, which impede free water diffusion and mimic the restricted diffusion pattern of aggressive or malignant lesions.

Epidermoid cysts, also termed epidermal inclusion cysts, form a further pitfall (Figure 25). Their contents are not simple fluid: laminated keratin, cholesterol and proteinaceous debris raise viscosity and impede free water motion, producing convincing restricted diffusion in a wholly benign lesion. The relevant point for musculoskeletal practice is that restriction here reflects the physical properties of the cyst contents rather than cellularity and that a subcutaneous or intramuscular lesion with restricted diffusion, a thin non-nodular wall and no solid enhancing component is more likely to be an epidermoid cyst than a malignancy.

### 12.6. Cellular Haematopoietic Marrow: Red Marrow Versus Infiltration

Reconverted or hyperplastic haematopoietic marrow is the most frequent source of false positive whole-body DWI in patients under investigation for myeloma or skeletal metastasis. The mechanism is not artefactual: red marrow is genuinely more cellular and more water-rich than yellow marrow and therefore genuinely restricts relative to it (Table 4, row 9). The signal is real; the inference of malignancy is not. The discriminator is not the diffusion data at all but the fat content, and the case illustrated in Figure 11 demonstrates the resolution: focal high DWI signal with apparently restricted diffusion in several thoracic vertebrae, in a patient whose Dixon fat fraction map confirms preserved marrow fat above the 20% threshold for significant marrow replacement, and whose T1-weighted signal remains higher than that of the adjacent intervertebral disc. The practical rule is that no focal marrow lesion on whole-body DWI should be called without paired fat fraction and T1-weighted correlation and that patients with chronic anaemia, a smoking history, obesity, or recent haematopoietic stimulation should be assumed to have reconverted marrow until proven otherwise.

### 12.7. Sampling and Quantification: Minimum Versus Mean ADC

ADC Overlap: Benign and malignant soft tissue masses show substantial ADC overlap, precluding reliable universal cut-offs. Minimum ADC tends to correlate more closely with histological grade in heterogeneous tumours because it samples the most cellular focus [42,55]; as discussed in Section 2.3, however, this biological relevance does not make it a more robust measurement—being an extreme value statistic, minimum ADC is in fact more sensitive to noise, single outlying pixels and ROI placement, so both metrics should be recorded and the metric used stated explicitly.

Postsurgical Assessment: Conventional MRI is limited postoperatively due to enhancing scar tissue. DWI adds specificity: Del Grande et al. demonstrated that a hypointense mass on ADC maps increased specificity for residual disease or recurrence from 52% to 97% (sensitivity 60%) [11].

## 13. Future Directions and Emerging Applications

The evolution of DWI in MSK radiology is increasingly directed towards its integration as a quantitative biomarker within multiparametric and radiomics-based frameworks. Radiomics and machine learning approaches applied to ADC maps and high-b-value DWI images are being explored for automated tumour grading, prediction of treatment response, and histological subtype classification, with a recent systematic review reporting promising but heterogeneous performance across soft tissue sarcoma studies [68]. DWI-derived radiomic nomograms have, for example, been applied to predict neoadjuvant chemotherapy response in osteosarcoma [69]. AI-based integration of DWI with conventional MRI, dynamic contrast-enhanced perfusion maps, and PET may yield multiparametric diagnostic and prognostic indices that surpass any single modality, although these approaches remain investigational and require external validation before clinical adoption [68].

A recurring obstacle to quantitative ADC analysis is that region-of-interest (ROI) placement remains manual and operator-dependent (Section 2.3) and is a principal source of measurement variability. Automated, deep learning-based segmentation offers a route to more reproducible ROI definition. Robust multiclass segmentation models are already well established for musculoskeletal CT; for example, multiclass learning approaches for skeletal muscle segmentation [70] and musculoskeletal segmentation models with uncertainty estimation that generalise across datasets [71]. Although deep learning segmentation for MSK MRI is comparatively less mature, translation of these cross-modality techniques to DWI could reduce the manual and variable component of ADC quantification.

Beyond sequence-based mitigation of susceptibility artefacts (multi-shot EPI, RESOLVE; Section 11), emerging post-acquisition computational methods, including deep learning-based distortion correction algorithms for echo-planar diffusion data, offer a complementary route to reducing geometric distortion and may further improve the reliability of quantitative DWI [72].

In sarcoma management, DWI-derived ADC changes are being evaluated as surrogate endpoints for histological response in neoadjuvant chemotherapy trials, potentially reducing reliance on repeat histological sampling of the tumour after neoadjuvant treatment as the reference standard for response [17,69]. Technical advances in DWI acquisition continue to expand diagnostic quality in anatomically challenging regions. Reduced field-of-view (rFOV) techniques including ZOOMit and iZOOM provide high-resolution DWI with reduced geometric distortion in small anatomical structures such as the foot, ankle, peripheral nerves, and wrist [26]. Readout-segmented EPI (RESOLVE) divides k-space readouts into multiple segments, reducing echo spacing and therefore geometric distortion and blurring relative to single-shot EPI; it is a multi-shot, two-dimensional technique rather than a three-dimensional acquisition, and its benefit lies in improved in-plane resolution and reduced susceptibility artefacts rather than in volumetric reformatting [3,40].

Standardisation of b-value protocols, phantom-based ADC calibration, and cross-platform harmonisation are prerequisites for DWI to achieve its full potential as a quantitative imaging biomarker in multicentre trials and routine clinical practice [3,8]. The role of WB-MRI with DWI in paediatric oncology surveillance, cancer predisposition syndromes, and monitoring of rare genetic myopathies and muscular dystrophies is an area of active investigation [32,59].

Diffusion is not the only quantitative adjunct available in musculoskeletal practice, and a multiparametric future is unlikely to rest on MRI alone. Shear wave elastography provides ultrasound-based quantification of tissue stiffness and biomechanical properties in tendons, ligaments, skeletal muscle, peripheral nerves and selected soft tissue lesions, and it has been explored for detecting early biomechanical alteration, grading disease severity, and monitoring response to treatment or rehabilitation [73]. Stiffness and diffusivity report on different physical properties of the same tissue, and the combination may prove more informative than either alone, particularly in muscles and nerves, where ADC alone has so far shown limited specificity. Like DWI, however, shear wave elastography currently lacks cross-platform standardisation, and the two techniques share the problem of threshold transferability, as discussed in Section 2.3.

## 14. Conclusions

DWI has evolved from a research-oriented sequence into a widely used adjunct in musculoskeletal MRI practice, though it is not yet an indispensable one, and the limitations of standardisation, reproducibility, artefacts and incomplete validation discussed throughout this review argue against describing it as such. Its capacity to provide qualitative and quantitative information on tissue microstructure complements conventional morphological sequences across tumour characterisation, infection, inflammatory conditions and systemic skeletal disease, but the strength of the supporting evidence varies considerably between these settings (Table 5). The ADC, while not a stand-alone diagnostic test, adds objective and reproducible data on tissue cellularity that supports clinical decision-making.

The organising claim of this review is that DWI in musculoskeletal practice should be read compositionally. The ADC is not a cellularity meter, and the lesions that expose this most sharply are precisely those in which the sequence appears to fail: the myxoid sarcoma that does not restrict, the epidermoid cyst that does, the gouty tophus with a spuriously low ADC in the absence of DWI hyperintensity, the haematoma indistinguishable from an abscess, and the hyperplastic red marrow indistinguishable from infiltration. Read as compositional evidence rather than as caveats, each of these becomes informative: the ADC ghost sign in osteomyelitis, the T2 black-through effect in haemosiderin-laden lesions, the ageing signature of venous thrombus, and the divergent ADC profiles of fibrous and myxoid matrices are not limitations to be managed but findings to be used. As technical innovation continues and multiparametric frameworks incorporating DWI, radiomics and artificial intelligence mature, this compositional reading, rather than any single ADC threshold, is what will allow DWI to serve as a dependable imaging biomarker for diagnosis, treatment monitoring and prognostication in musculoskeletal radiology. Importantly, while absolute ADC thresholds may vary with acquisition, scanner and analysis methodology, qualitative assessment of visually evident restricted diffusion can be more robust across different malignant tumour types and other musculoskeletal lesions, including abscesses.

### Key Points

The DWI signal is the joint product of water mobility, intrinsic T2 relaxation and mobile proton density; where these diverge, the discordance is specific to lesion composition and is diagnostically informative rather than merely a pitfall (Table 4) [3,8,12].

DWI measures water diffusivity based on Brownian motion; restricted diffusion (high DWI signal with low ADC) reflects impeded water motion, most often from high cellularity, as in many malignancies, or from viscous proteinaceous material, as in pus and in the contents of epidermoid cysts [1,2,3]. Coagulative necrosis with preserved viscous debris may also restrict; this should not be confused with treatment-induced necrosis, which loosens tissue architecture and characteristically raises the ADC.

Applications differ substantially in evidentiary maturity (Table 5). Only whole-body DWI for myeloma staging and response assessment is guideline-endorsed. Soft tissue abscess detection, tumour characterisation and vertebral fracture assessment rest on consistent evidence from multiple cohorts but without transferable ADC thresholds. The ADC ghost sign is highly specific but has a sensitivity of only approximately 20% and is a rule-in finding only [3,8,25].

DWI enables treatment response monitoring through rising ADC values following effective chemotherapy or radiotherapy, providing a contrast-free surrogate for histological necrosis in soft tissue tumours and bone sarcomas [17].

Whole-body DWI is guideline-endorsed for myeloma staging and response assessment under MY-RADS, with supporting prospective multicentre data. Its use in metastatic workup is supported by smaller cohorts, and its application to NF1 surveillance and paediatric cancer predisposition monitoring remains investigational, with no outcome data [18,27,59].

Opportunistic detection of venous thrombus on musculoskeletal DWI is a useful incidental finding worth reporting.

Composition-related discordance follows predictable patterns: myxoid, chondroid matrix and simple fluid raise the ADC; hemosiderin, dense collagen and crystalline or mineralised material lower both DWI and ADC signal; and viscous keratinaceous contents restrict in the absence of hypercellularity [3,8,10,12].

Technical pitfalls such as T2 shine-through, T2 black-through and characteristic ADC pitfalls of certain tumour subtypes should be actively recognised and leveraged as diagnostic tools rather than treated purely as limitations [3,8,12].

DWI should be used as a supplementary functional imaging technique to conventional MRI and interpreted in clinical context; it should never be assessed in isolation [3,8].

## Figures and Tables

**Table 1:** Summary of the principal studies underpinning the applications discussed in this review.

Study (Ref)	Design	*n*	Field/b-Values (s/mm^2^)	Reference Standard	Principal Findings
Razek 2012 [9]	Prospective, single-centre	37	1.5 T/[0.500 and 1000]	Histopathology	Mean ADC cut-off 1.34 × 10^−3^ for excluding malignancy; accuracy 91%, sens 94%, spec 88%
Subhawong 2013 [10]	Retrospective, single-centre	36	3.0 T/[50, 400 and 800]	Histopathology/follow-up	Mean ADC > 1.7 and min ADC > 1.3: sens 40%, spec 92%; haematoma min 0.56, mean 0.82
Del Grande 2014 [11]	Retrospective, single-centre	37	3.0 T/[50, 400 and 800	Histopathology/follow-up	Min ADC ≤ 0.8 only independent predictor of malignancy (sens 75%, spec 73%); hypointense ADC mass raised specificity 52 → 97%
Chhabra 2019 [12]	Retrospective	51	1.5/3.0 T/[50, 400, and 600–800]	Histologic grade	DWI/ADC correlates with histologic grading of soft tissue malignancy
Ogawa 2019 [7]	Prospective	43	3.0 T/>1000 (DKI)	Histopathology	DKI metrics differentiate malignant from benign MSK tumours
Einarsdóttir 2004 [13]	Prospective, small cohort	29	1.5 T/[0 and 600]	Histologic necrosis	Post-radiation ADC increase correlated with 40–60% tumour necrosis
Dudeck 2008 [14]	Prospective	23	1.5 T/[0, 500, and 1000]	Tumour volume/response	Inverse relationship between ADC and tumour volume after treatment
Soldatos 2016 [15]	Prospective	23	3.0 T/[50, 400, and 800]	Histologic response	Min ADC > 2.0 × 10^−3^ indicates good response: sens 100%, spec 61%
Suh 2018 [16]	Systematic review and meta-analysis	Twelve eligible studies (748 lesions, 661 patients)	Mixed	Histopathology/follow-up	Pooled sensitivity 89–92%, specificity 87–91% for benign vs. malignant vertebral lesions; thinner slices improved specificity
Yuan 2022 [17]	Systematic review and meta-analysis	13 studies [303 patients]	Mixed	Histologic necrosis	DWI effective for neoadjuvant chemotherapy response in osteosarcoma
Zhang 2021 [18]	Prospective cohort	114	3.0 T/[50 and 800]	Progression/survival	Baseline marrow ADC an independent predictor of progression and death in newly diagnosed myeloma
Messiou 2019 (MY-RADS) [19]	Consensus guideline	n/a	1.5/3.0 T/50–100 and 800–900	Expert consensus	Defines ADC bands (normal < 600–700; viable 700–1400; treated ≥ 1400 µm^2^/s) and five-point RAC scale
Giles 2014 [20]	Prospective	40	1.5 T/[50 and 900]	Clinical/serologic response	ADC rise in 95% of responders vs. decline in all non-responders
Moreau 2017 (IMAJEM) [21]	Prospective multicentre RCT sub-study	134	Mixed	PFS/OS	Conventional MRI normalised in only 3–11%; no predictive value for PFS/OS—supports functional DWI
Messiou 2025 (iTIMM) [22]	Prospective multicentre	70	Mixed/per MY-RADS	Serology + marrow MRD	MY-RADS WB-MRI gives prognostic information independent of serology and MRD
Chun 2018 [23]	Prospective comparative	119	3.0 T/[0 and 800]	Surgery/microbiology	DWI comparable to CE-MRI for abscess; mean ADC abscess 0.88, phlegmon 2.2, oedema 3.1 × 10^−3^
Wudhikulprapan 2024 [24]	Systematic review and meta-analysis	5 studies 187 patients	Mixed	Bone biopsy/culture	DWI and DCE-MRI performance for diabetic foot osteomyelitis
He 2025 [25]	Retrospective	178	1.5/3.0 T/b value not available	Histopathology/culture	ADC ghost sign sensitivity 19.5%, specificity 94.5% vs. T1 penumbra 3.7%/98.9%, T1 ghost 9.8%/97.8%
Xia 2024 [26]	Prospective comparative	43	1.5/3.0 T/[50, 400, and 800]	Clinical/surgical	ZOOMit (rFOV) DWI superior to conventional DWI in foot/ankle infection
Ristow 2024 [27]	Prospective intra-individual	34	3.0 T/[0, 10, 20, 30, 50, 70, 100, 300, 400, 600, 800]	Histopathology	DWI vs. PET for discriminating benign, atypical and malignant PNST in NF1
Ahlawat 2018 [28]	Retrospective	48	1.5/3.0 T/[50, 400, and 800]	Histopathology	Min ADC ≤ 1.0 × 10^−3^ indicator of malignancy in PNST
Demehri 2014 [29]	Prospective, initial experience	31	3.0 T/[50, 400, and 800]	Histopathology	Size >4.2 cm plus low ADC supports malignancy in PNST
Qi 2008 [30]	Prospective feasibility	8	1.5 T/[0, 10, 20, 30, 40, 50, 60, 70, 80, 90, 100, 150, 200, 250, 300, 350, 400, 450, 500, 600, 700, 800, 900, and 1000]	Clinical diagnosis (IIM)	Elevated ADC/D in inflamed muscle; lowest ADC in fatty-replaced muscle
Faruch 2019 [31]	Prospective	60	1.5 T/[50 and 800]	Clinical/STIR grade	ADC correlates with muscle oedema grade; detects oedema before STIR change
Saran 2025 [32]	Case–control (DTI)	43	3.0 T/[500 and 1000]	Clinical diagnosis (IIM)	Higher ADC, FA and eigenvalues in IIM vs. controls (vastus lateralis)
Guggenberger 2013 [33]	Prospective, multi-scanner	16	3.0 T/[0 and 1200]	Healthy volunteers	Normal median-nerve ADC ≈ 1.01 ± 0.13 × 10^−3^; assesses cross-scanner agreement
Haakma 2017 [34]	Case–control	30	3.0 T/[0 and 800]	Clinical/electrophysiology	Nerve thickening and altered diffusion in multifocal motor neuropathy
Gi 2020 [35]	Retrospective/experimental	8	1.5/3.0 T/b value not available	Histology/clinical timing	Acute DVT ADC 0.56 ± 0.17 vs. non-acute 0.22 ± 0.12 × 10^−3^; sensitivity 93%, specificity 90%
Oka 2008 [36]	Retrospective	37	1.5 T/[500 and 1000]	Histopathology	Chronic expanding haematoma mean ADC 1.55 ± 0.12 vs. malignant STT 0.92 ± 0.14 × 10^−3^ (*p* < 0.01)
Donners 2022 [37]	Retrospective	43	1.5 T/[50 and 900]	Biopsy yield/NGS	Multiparametric bone MRI improves CT-guided biopsy target selection and yield

**Table 2:** Provenance of some of the principal ADC thresholds highlighted in this review.

Threshold (Application)	Study (Ref)	Population	Acquisition (Field/b-Values s/mm^2^)	ROI Strategy	Reference Standard	Validation Status
Min ADC < 1.0 × 10^−3^ (soft tissue malignancy)	Del Grande 2014 [11]	37	3.0 T/[50, 400, and 800]	Min-ADC ROI, viable tissue	Histopathology/follow-up	Single-cohort; not externally validated
Min ADC > 2.0 × 10^−3^ (sarcoma treatment response)	Soldatos 2016 [15]	23	3.0 T/[50, 400, and 800]	Min-ADC ROI	Histologic response	Single-centre prospective; unvalidated
Min ADC ≤ 1.0 × 10^−3^ (MPNST)	Ahlawat 2018 [28]	48	1.5/3.0 T/[50, 400, and 800]	Min-ADC ROI	Histopathology	Retrospective; unvalidated
Marrow bands <600–700/700–1400/≥1400 µm^2^/s (MY-RADS)	Messiou 2019 [19]	Consensus (n/a)	1.5/3.0 T/50–100 and 800–900	Per MY-RADS	Expert consensus	Guideline-defined; guideline-endorsed
ADC 0.56 vs. 0.22 × 10^−3^ (DVT ageing)	Gi 2020 [35]	8	1.5/3.0 T/b value not available	Thrombus ROI	Histology/clinical timing	Single-centre; SDs overlap; unvalidated

**Table 3:** Indicative reference ADC values for key musculoskeletal structures [8,45]. These are representative figures drawn from the cited sources and are not transferable cut-offs; see Section 2.3 for the acquisition and measurement factors that shift them.

Structure	Normal ADC Value (×10^−3^ mm^2^/s)	Clinical Relevance
Yellow bone marrow	0.1–0.3	Hypointense on DWI; hydrophobic fat cells
Red (cellular) marrow	0.4–0.7	Higher water and cellular contents; needs to be distinguished from marrow-infiltrating pathologies
Articular cartilage	1.0–1.1	Elevated ADC indicates early cartilage damage
Peripheral nerve	1.2–1.3	Reduced FA/elevated ADC in neuropathy
Skeletal muscle (normal)	1.3–1.5	Elevated ADC in active myositis/denervation
Simple fluid/water	2.5–4.0	T2 shine-through in cystic lesions

**Table 4:** Lesion composition and its diffusion signature: dominant substrate, physical mechanism, expected DWI and ADC behaviour, interpretive consequence, and illustrative case [3,8,10,11,12,35,36,46].

Dominant Substrate	Physical Mechanism	DWI Signal	ADC	Interpretive Consequence	Figure
Chondroid matrix	Abundant water-rich hyaline cartilage and myxoid matrix, prolonged T2 relaxation time. Malignant cells are often dispersed rather than being densely packed to show restricted diffusion.	High	High	Malignant chondroid tumours/chondrosarcomas may not restrict; ADC cannot grade this subtype. Helpful in differentiating them from chordoma, which similarly have high T2 signal but show restricted diffusion [47].	5, 6
Cellular haematopoietic (red) marrow	High cellularity and water content in reconverted marrow.	High	Low	Mimics diffuse plasma cell infiltration in myeloma; resolved by Dixon fat fraction (20% threshold).	11
Purulent material	High cellularity and viscous inflammatory debris.	High	Low	True restriction; permits abscess diagnosis without contrast.	15, 17
Organising thrombus [48]	Time-dependent fibrosis and progressive water loss.	Acute high; non-acute low	Acute 0.56 ± 0.17; non-acute 0.22 ± 0.12 × 10^−3^ mm^2^/s	Thrombus age determines suitability for thrombolysis.	18, 19
Myxoid extracellular matrix	Abundant water-rich extracellular matrix, composed predominantly of glycosaminoglycans with low collagen density, resulting in increased water mobility, prolonged T2 relaxation time [49,50].	High (shine-through)	High, often >2.0 × 10^−3^ mm^2^/s	Malignant myxoid tumours may not restrict; ADC cannot grade this subtype.	20, 21
Blood degradation products	Intracellular methaemoglobin, raised viscosity, reduced extracellular water mobility.	Variable	Low in acute and early subacute; rises with liquefaction	Haematoma mimics sarcoma and abscess; ADC is a function of haemorrhage age.	23
Crystalline and mineralised material	Low proton density, calcification, fibrosis.	Low	Spuriously low	Low ADC without corresponding DWI hyperintensity is not true restriction.	24
Keratin, cholesterol, proteinaceous debris [51]	High viscosity impedes water motion.	High	Low	Benign cystic lesions may convincingly restrict.	25
Hemosiderin [52]	Paramagnetic T2 shortening.	Low	Low	T2 black-through is a positive marker of haemosiderin-laden disease (Giant cell tumour of bone and tendon sheath, PVNS).	—
Dense fibrocollagenous matrix	Low mobile proton density, restricted free water.	Low	Low	Mature desmoid-type fibromatosis. More cellular desmoids have variable appearance on T2, DWI and ADC images.	—
Simple fluid; synovial osteochondromatosis	Free water with long T2 relaxation.	High (shine-through)	High	Separates synovial osteochondromatosis from PVNS.	—

**Table 5:** Evidentiary maturity of the musculoskeletal DWI applications reviewed. Tier 1 = supported by guidelines or prospective multicentre evidence; Tier 2 = supported by consistent evidence from multiple independent cohorts but not standardised, with no transferable threshold; Tier 3 = promising adjunct supported by small, single-centre or heterogeneous studies, thresholds not externally validated; Tier 4 = experimental.

Application	Evidence Tier	Basis and Principal Limitation	Section
Whole-body DWI: myeloma staging and response assessment	Tier 1	MY-RADS consensus guidance; prospective multicentre iTIMM data; recommended standard in some national pathways. ADC response thresholds still require local calibration.	Section 5.1
Soft tissue abscess detection	Tier 2	Consistent evidence from several independent cohorts, including comparison against contrast-enhanced MRI. Particularly valuable where contrast is contraindicated. No validated ADC cut-off.	Section 6.1
Soft tissue tumour: benign versus malignant	Tier 2	Multiple single-centre series and meta-analytic data, but substantial ADC overlap between groups and no universally transferable threshold. Compositional exceptions are systematic, not rare.	Section 4.1
Bone lesion and vertebral compression fracture characterisation	Tier 2	Meta-analysis reports pooled sensitivity of 89–92% and specificity of 87–91%. Thresholds vary between studies and with acquisition protocol.	Section 5.3 and Section 5.4
Treatment response in soft tissue and bone sarcoma	Tier 2 to 3	Direction of ADC change after effective treatment is consistent across studies; specific cut-offs derive from small cohorts and, in several instances, were derived and tested in the same population.	Section 4.3 and Section 5.5
Postsurgical residual or recurrent disease	Tier 3	Single-centre series showing improved specificity over conventional MRI. Not externally validated.	Section 4.4
ADC ghost sign in osteomyelitis	Tier 3	Single retrospective series. Specificity of approximately 95% but sensitivity of approximately 20%; a rule-in sign only, with negligible negative predictive value.	Section 6.2
Peripheral nerve sheath tumour characterisation and DTI tractography	Tier 3	Small heterogeneous series. Benign schwannomas with cellular Antoni A regions overlap malignant lesions on minimum ADC.	Section 4.5
Whole-body DWI in NF1 and paediatric cancer predisposition surveillance	Tier 3	Small cohorts; the radiation-free advantage is compelling, but no outcome or survival data exist.	Section 7
Inflammatory myopathy	Tier 3	Small heterogeneous studies; progressive fatty infiltration confounds ADC in chronic disease.	Section 8
Peripheral neuropathy and plexopathy	Tier 3	Small series; low signal-to-noise ratio in small-calibre nerves and limited specificity against other neuropathies.	Section 9
Opportunistic detection of deep vein thrombosis	Tier 3	Detection as an incidental finding is plausible and clinically useful; ADC-based thrombus ageing rests on a single small cohort without external validation and does not determine management.	Section 10
Radiomics, machine learning and multiparametric AI indices	Tier 4	Exploratory. No prospective validation, and cross-platform ADC variability is a fundamental obstacle to generalisable models.	Section 13

**Table 6:** DTI tractography fibre patterns and ADC characteristics in peripheral nerve sheath tumours [27,29,57].

Tumour Type	Fibre Appearance on Tractography	ADC Characteristic
Schwannoma	Displaced fibres around lesion	Higher ADC (benign)
Neurofibroma	Splayed fibres traversing lesion	Higher ADC (benign)
MPNST	Infiltrated/disrupted fibres	Low ADC (<1.0 × 10^−3^ mm^2^/s)

**Table 7:** DWI and ADC characteristics across the spectrum of musculoskeletal infections [3,8,23,25,65].

Condition	DWI Signal	ADC Map	ADC (×10^−3^ mm^2^/s)
Intraosseous abscess	Bright	Dark (restricted)	0.6–1.0
Soft tissue abscess	Bright	Dark (restricted)	0.6–1.1
Active osteomyelitis (+ghost sign)	Bright	High in bone	1.1–1.6
Cellulitis	Bright	Mildly dark	1.2–2.0
Bland soft tissue oedema	Bright	Bright (T2 shine-through)	2.0–3.0
Reactive marrow oedema	Variable	Variable	1.4–1.9

**Table 8:** ADC profile across muscle states in inflammatory myopathy [30,31,32].

Muscle State	DWI Signal	ADC Finding	Clinical Significance
Normal	Low/Dark	Baseline (1.3–1.5 × 10^−3^ mm^2^/s)	Reference range
Inflamed (active IIM)	Elevated	Elevated ADC and D	Active inflammation
Fatty replacement (chronic)	Variable	Spuriously low ADC	Technical pitfall

**Table 9:** DWI and ADC characteristics for differentiating acute from non-acute deep vein thrombosis [35]. Values derive from a single, small cohort and are indicative only; they have not been externally validated and should not be used as thresholds in clinical practice.

Thrombus Stage	Water Content	DWI Signal	ADC Value (×10^−3^ mm^2^/s)
Acute (<14 days)	Higher (metHb T2 shortening)	High (Bright)	0.56 ± 0.17
Non-acute (>14 days)	Lower (organised thrombus)	Low (Dark)	0.22 ± 0.12

## Data Availability

No new data were created or analysed in this study. Data sharing is not applicable to this article.
